# A novel weighting method to remove bias from within-subject exposure dependency in case-crossover studies

**DOI:** 10.1186/s12874-021-01408-5

**Published:** 2021-10-17

**Authors:** Kiyoshi Kubota, Thu-Lan Kelly, Tsugumichi Sato, Nicole Pratt, Elizabeth Roughead, Takuhiro Yamaguchi

**Affiliations:** 1 NPO Drug Safety Research Unit Japan|, 6-2-9-2F, Soto-Kanda, Chiyoda-ku, Tokyo, 101-0021 Japan; 2 Department of Pharmacy, Tokyo University of Science, Chiba, Japan; 3 Quality Use of Medicines Pharmacy Research Centre, Clinical and Health Sciences, University of South Australia, Adelaide, Australia; 4 Division of Biostatistics, Tohoku University Graduate School of Medicine, Miyagi, Japan

**Keywords:** Case-crossover study, bias, Autocorrelation

## Abstract

**Background:**

Case-crossover studies have been widely used in various fields including pharmacoepidemiology. Vines and Farrington indicated in 2001 that when within-subject exposure dependency exists, conditional logistic regression can be biased. However, this bias has not been well studied.

**Methods:**

We have extended findings by Vines and Farrington to develop a weighting method for the case-crossover study which removes bias from within-subject exposure dependency. Our method calculates the exposure probability at the case period in the case-crossover study which is used to weight the likelihood formulae presented by Greenland in 1999. We simulated data for the population with a disease where most patients receive a cyclic treatment pattern with within-subject exposure dependency but no time trends while some patients stop and start treatment. Finally, the method was applied to real-world data from Japan to study the association between celecoxib and peripheral edema and to study the association between selective serotonin reuptake inhibitor (SSRI) and hip fracture in Australia.

**Results:**

When the simulated rate ratio of the outcome was 4.0 in a case-crossover study with no time-varying confounder, the proposed weighting method and the Mantel-Haenszel odds ratio reproduced the true rate ratio. When a time-varying confounder existed, the Mantel-Haenszel method was biased but the weighting method was not. When more than one control period was used, standard conditional logistic regression was biased either with or without time-varying confounding and the bias increased (up to 8.7) when the study period was extended. In real-world analysis with a binary exposure variable in Japan and Australia, the point estimate of the odds ratio (around 2.5 for the association between celecoxib and peripheral edema and around 1.6 between SSRI and hip fracture) by our weighting method was equal to the Mantel-Haenszel odds ratio and stable compared with standard conditional logistic regression.

**Conclusion:**

Case-crossover studies may be biased from within-subject exposure dependency, even without exposure time trends. This bias can be identified by comparing the odds ratio by the Mantel-Haenszel method and that by standard conditional logistic regression. We recommend using our proposed method which removes bias from within-subject exposure dependency and can account for time-varying confounders.

**Supplementary Information:**

The online version contains supplementary material available at 10.1186/s12874-021-01408-5.

## Background

The case-crossover design has been widely used since it was proposed in 1991 [1]. The design has been used in various fields including pharmacoepidemiology [2], occupational epidemiology [3], studies on traffic safety [4] and air pollution health effects [5, 6]. In case-crossover studies, individuals who have experienced the outcome (cases) act as their own controls by including one or more periods before the onset of the outcome. The period including the outcome is the case period, while period(s) prior to the case period act as the controls. The number of control periods can be large: for example, in the original article [1], one analysis involved 8766 control periods. The effect of the exposure should be brief; exposure in any period should affect the outcome in that period only, without any ‘carryover effect’ [1, 6, 7]. In addition, in case-crossover studies, the rate of outcome occurrence is usually assumed to be unchanged during exposed or unexposed periods, respectively. However, like other case-only studies, the case-crossover study has an advantage that the effect of time-invariant confounders is automatically controlled because the case period is compared with control period(s) of the same individual [7–9]. The case-crossover study also has unique characteristics. For example, the original unidirectional case-crossover study does not include periods after the outcome occurs. Thus, there is no bias due to the outcome influencing future exposures or future observation periods, unlike other case-only studies such as self-controlled case series [10–12].

However, case-crossover studies are susceptible to at least two types of major biases. The first is bias due to time trends in the exposure which can be removed using a variant of case-crossover studies, the case-time-control design [9, 13, 14]. The second is bias due to within-subject exposure dependency or autocorrelation in an individual’s exposure history [6, 15, 16]. This bias is particularly important in pharmacoepidemiology because drug use on 1 day is rarely independent from use in the preceding days. However, the potential for this bias has had little attention in the pharmacoepidemiology literature, and standard conditional logistic regression has been used without assessing whether bias due to within-subject exposure dependency exists in many case-crossover studies [17–21]. This may be in part because this bias has been considered rather minor when exposure is stationary [15].

In this paper, we show that within-subject exposure dependency may produce unignorably large bias even if exposure is stationary. We also describe how to remove bias from within-subject exposure dependency, when time trends in the exposure do not exist.

### Motivating examples

Our motivation for investigating bias in case-crossover studies due to within-subject exposure dependency was a hypothetical drug treatment with a specific cyclic exposure pattern, where standard conditional logistic regression may produce bias. We also outline real-world pharmacoepidemiological studies in Japan and Australia where our proposed method is used.

### Drug treatment with specific exposure pattern

We illustrate our motivating example using a drug treatment with a cyclic pattern consisting of two periods of treatment followed by one period with no treatment. The exposure pattern clearly shows autocorrelation, with the probability of exposure in one treatment period dependent on previous periods. We show that estimates of the odds ratio for the exposure-outcome association may be biased, depending on the number of control periods chosen.

In a hypothetical unidirectional case-crossover study with 4 periods (one case period and 3 control periods), only 3 exposure patterns are possible. If exposure and non-exposure are indicated by 1 and 0, respectively, the 3 exposure patterns are (1101), (1011), and (0110). Therefore, subjects with an exposed case period can have only 1 unexposed and 3 exposed periods, while those with unexposed case period can have only 2 unexposed and 2 exposed periods. In such a case-crossover study, when the true odds ratio (OR) is 4.0, the OR is underestimated as 3.2 using standard conditional logistic regression for matched case-control studies.

However, if there are two control periods, the possible exposure patterns are (110), (101), and (011). In this case, irrespective of whether the case period is exposed or unexposed, all subjects have 1 unexposed and 2 exposed periods and there is no bias in the estimate of the OR. Similarly, there is no bias when the total number of periods (including the case period) is an integral number of 3 (the cyclic pattern).

### Overview of real-world examples

We describe two real world pharmacoepidemiological studies which will be analyzed by our proposed method. The first is an investigation into a previously reported association between celecoxib and peripheral edema [22] using Japanese data from 25 corporate-type health insurance plans [23]. From 99,821 new users of celecoxib aged between 20 and 74 years from May 2013 to April 2018, who used celecoxib after at least 180 days of non-use, we selected 311 cases who experienced peripheral edema and had both exposed and unexposed days during an 84-day study period.

The second example is an Australian study of the association between hip fracture and psychoactive medicines [24, 25]. The data were obtained from the Australian Department of Veterans’ Affairs administrative claims database. Psychoactive medicines included benzodiazepines, selective serotonin re-uptake inhibitors (SSRIs), opioids, antipsychotics and tricyclic antidepressants. The hip fracture cases were 8828 patients aged over 65 years who were hospitalized between 2009 and 2012. A previous case-crossover study of this cohort found an increased risk of hip fracture for opioids, SSRIs and antipsychotics [24]. A related case-control study found an association between hip fracture and SSRIs when used concurrently with other psychoactive medicines [25].

## Methods

To avoid bias due to within-subject exposure dependency, the likelihood for estimating the odds ratio for the exposure-outcome association may be modified. At least two approaches are effective to modify the likelihood and both involve two-step weighting procedures. In the first approach, the probability of each exposure permutation is estimated in Step 1 and used as weights in the likelihood in Step 2. In the special case where this probability is the same for every permutation (“global exchangeability”), the modified likelihood is equivalent to that for the standard conditional logistic regression.

In this paper, we propose another approach that can be used if “pairwise exchangeability” is assumed. Pairwise exchangeability holds where there are no time trends in the exposure i.e., the proportion exposed in the population is stationary over the study period [15]. In this scenario, the probabilities that the case period is exposed and unexposed are estimated in Step 1 and used as weights in Step 2. When there is pairwise exchangeability, an unbiased OR can be obtained by the Mantel-Haenszel method as well.

### BIAS due to conditional logistic regression for case-crossover studies

In 2001, Vines and Farrington proposed the likelihood for case-crossover studies as [15]:

1 $$L=\prod_{i=1}^N\frac{\exp \left(\beta {x}_{i0}\right){\sum}_{\kappa \ast }P\left\{{X}_{i0}={x}_{i\kappa \ast (0)},---,{X}_{i M}={x}_{i\kappa \ast (M)}\right\}}{\sum_{\kappa}\exp \left(\beta {x}_{i\kappa (0)}\right)P\left\{{X}_{i0}={x}_{i\kappa (0)},---,{X}_{i M}={x}_{i\kappa (M)}\right\}}$$

where *X*_*i*0_ is the exposure level at the case period (m = 0) and *X*_*im*_ is the exposure level at the m-th control period at t (*t* =  − *m*: m = 1, 2, −-, M), *x*_*i*0_ denotes the observed exposure level at the case period and *x*_*im*_ denotes the observed exposure level at the m-th control period, the sum in the denominator ranges over all permutations of κ of the integers {0, 1, −--, M} and the sum in the numerator ranges over the subset of the permutations for which *x*_*iκ*(0)_ = *x*_*i*0_ of the individual i. In Eq. (1), *X*_*i*0_ may be a binary exposure variable but can be a multi-level exposure variable or a vector of an exposure and time-varying confounders. When *X*_*im*_ denotes a binary exposure, the denominator in Eq. (1) becomes $\exp \left(\beta \right){\sum}_{\kappa_1}P\left({X}_{i0}=1,{N}_{exposed}=k\right)+{\sum}_{\kappa_0}P\left({X}_{i0}=0,\kern0.5em {N}_{exposed}=k\right)$ where *N*_*exposed*_ is the total number of exposed (case and control) periods, given by ${N}_{exposed}={\sum}_{m=0}^M{X}_{im}$ and ${\sum}_{\kappa_1}P\left({X}_{i0}=1,{N}_{exposed}=k\right)$ is the sum of probabilities for all the permutations of k exposed and (M + 1-k) unexposed periods where *X*_*i*0_ = 1, and ${\sum}_{\kappa_0}P\left({X}_{i0}=0,\kern0.5em {N}_{exposed}=k\right)$ is that where *X*_*i*0_ = 0. Data on *N*_*exposed*_ = *k* is informative only when the positivity (non-zero probability) condition is satisfied or ∑*P*(*X*_*i*0_ = 1, *N*_*exposed*_ = *k*) > 0 and ∑*P*(*X*_*i*0_ = 0 *N*_*exposed*_ = *k*) > 0. Otherwise, they do not contribute to the estimation of exp(*β*).

Let *OR*_*VF*_ be the estimate of exp(*β*) obtained from Eq. (1). The likelihood in Eq. (1) and *OR*_*VF*_ are in general different from the following likelihood for the standard conditional logistic (SCL) regression for individually matched case-control studies and its estimate, *OR*_*SCL*_.

2 $$L=\prod_{i=1}^N\frac{\exp \left(\beta {x}_{i0}\right)}{\sum_{j=0}^M\exp \left(\beta {x}_{ij}\right)}$$

Vines and Farrington showed that the likelihoods in Eqs. (1) and (2) are equivalent if P{*X*_*i*0_ = *x*_*i*0_, − − −, *X*_*iM*_ = *x*_*iM*_} = *P*{*X*_*i*0_ = *x*_*iκ*(0)_, − − −, *X*_*iM*_ = *x*_*iK*(*M*)_} for all permutations κ of {0, 1, −---, M}, that is, global exchangeability holds. For example, Eqs. (1) and (2) are equivalent when the exposure status in one period is independent from the status in any other periods and the exposure probability is the same in all of case and control periods (Appendix 1, Additional File 1). If global exchangeability does not hold, *OR*_*SCL*_ can be biased.

Vines and Farrington did not show explicitly how to estimate *P*{*X*_*i*0_ = *x*_*iκ*(0)_, − − −, *X*_*iM*_ = *x*_*iκ*(*M*)_} in Eq. (1). These probabilities may be estimated by the proportion of each permutation in the population which contain cases, or in samples representing the population such as time-controls in the case-time-control design proposed by Suissa [14]. In the next section, however, we introduce a different approach to remove the bias from within-subject exposure dependency by assuming that pairwise exchangeability is satisfied but global exchangeability may not necessarily hold.

### Weighting method to remove BIAS due to within-exposure dependency

#### Case-crossover studies with a binary exposure

When pairwise exchangeability, P{*X*_*i*0_ = 1, *X*_*im*_ = 0} = *P*(*X*_*i*0_ = 0, *X*_*im*_ = 1} is satisfied for a binary exposure in all control periods m (m = 1, 2, −-, M), the estimate of exp(*β*) using the Mantel-Haenszel method (*OR*_*MH*_) is unbiased whether within-subject exposure dependency exists or not [15]. In line with this finding, we will show that when pairwise exchangeability holds, the probabilities that the individual is unexposed (π_0_) and exposed (π_1_) at the case period, can be estimated from the cases in a case-crossover study (without requiring data from the population or time-controls). Once π_0_, π_1_, and the relative exposure probability π_10_ = π_1_ / π_0_ are estimated, the following likelihood for case-crossover studies proposed by Greenland [26] can be used to obtain an unbiased estimate of exp(*β*), defined as *OR*_*G*_:

3 $$\mathrm{L}=\prod_{i=1}^N\frac{\exp \left({\beta x}_{ic}\right){\pi}_{ic}}{\sum_k\exp \left(\beta {x}_k\right){\pi}_{ik}}=\prod_{i=1}^N\frac{\exp \left({\beta x}_{ic}\right){\pi}_c}{\sum_k\exp \left(\beta {x}_k\right){\pi}_k}=\prod_{i=1}^N\frac{\exp \left({\beta x}_{ic}\right){\pi}_{c0}}{\sum_k\exp \left(\beta {x}_k\right){\pi}_{k0}}$$

In the left-hand side of Eq. (3), *π*_*ik*_ is the probability that individual i has the k-th exposure level at the case period (k = 0, 1 for binary exposure), *π*_*ic*_ is *π*_*ik*_ observed, and *x*_*ic*_ is the exposure level observed when individual i has the outcome. In the middle of Eq. (3), subscript i is omitted in the exposure probabilities as *π*_*ik*_ is replaced by the expected value in the population in the current study. In the right-hand side of Eq. (3) *π*_*k*0_ = *π*_*k*_/*π*_0_. Eq. (3) stands for the model where *x*_*ik*_ is a binary variable, multi-level exposure variable, or a vector of the observed exposure and time-varying confounders.

For a binary exposure, the right-hand side of Eq. (3) can be rewritten as

4 $$\mathrm{L}=\prod_{i=1}^N\frac{\exp \left({\beta x}_{ic}\right){\pi}_{c0}}{1+\exp \left(\beta \right){\pi}_{10}}$$

where *x*_*ic*_ = 1 and *π*_*c*0_ = *π*_10_ = *π*_1_/*π*_0_ when the individual i was exposed at the case period and *x*_*ic*_ = 0 and *π*_*c*0_ = *π*_00_ = *π*_0_/*π*_0_ = 1 when unexposed. As Vines and Farrington showed [15], Greenland did not estimate *π*_*k*_ for case-crossover studies in Eq. (3) when within-subject exposure dependency exists.

We outline a novel weighting method using a modified version of Greenland’s likelihood. We propose that *π*_*k*_ can be estimated, with or without within-subject exposure dependency, by assuming pairwise exchangeability. Let P_*kl*[*m*]_ denote the joint probability that the subject has the k-th exposure level at the case period and has the *l*-th exposure level at the m-th control period:

5 $${\mathrm{P}}_{kl\left[m\right]}=\mathrm{P}\left({X}_0={x}_k,\kern0.5em {X}_m={x}_l\right)\kern0.50em \mathrm{m}=1,2,---,\mathrm{M}$$

In Eq. (5), *X*_0_ is the exposure status at the case period and *X*_*m*_ is the exposure status at the m-th control period. When the exposure variable is binary, both *X*_0_ and *X*_*m*_ have two levels (k = 0, 1 and *l* = 0,1) and pairwise exchangeability is equivalent to stationary exposure (no time trends): when the exposure process is stationary,  P_10[*m*]_ + P_11[*m*]_ = P_01[*m*]_ + P_11[*m*]_ and this relationship leads to the pairwise exchangeability condition P_10[*m*]_ = P_01[*m*]_ (m = 1, 2, −-, M).

Using conditional probabilities, pairwise exchangeability, P{*X*_*i*0_ = 1, *X*_*im*_ = 0} = *P*(*X*_*i*0_ = 0, *X*_*im*_ = 1} can be rewritten as:

6 $${\uppi}_1\mathrm{P}\left(\ {X}_m=0|{X}_0=1\right)={\uppi}_0\mathrm{P}\left(\ {X}_m=1|{X}_0=0\right)$$

where π_0_ = P(*X*_0_ = 0) and π_1_ = P(*X*_0_ = 1). When both sides of Eq. (6) are summed up over M control periods (m = 1, 2, −-, M), we obtain:

7 $${\uppi}_1{\sum}_{m=1}^M\mathrm{P}\left(\ {X}_m=0|{X}_0=1\right)={\uppi}_0{\sum}_{m=1}^M\mathrm{P}\left(\ {X}_m=1|{X}_0=0\right)$$

The quantity ${\sum}_{m=1}^M\mathrm{P}\left(\ {X}_m=0|{X}_0=1\right)$ can be estimated by the average number of unexposed control periods (where *X*_*m*_ = 0) in those exposed at the case period (*X*_0_ = 1). Similarly, the quantity ${\sum}_{m=1}^M\mathrm{P}\left(\ {X}_m=1|{X}_0=0\right)$ can be estimated as the average number of exposed control periods (*X*_*m*_ = 1) in those unexposed at the case period (*X*_0_ = 0). This average, defined as $\overline{PT_{10}}$ and $\overline{PT_{01}}$, respectively, can be written as:

8 $$\overline{PT_{10}}={\sum}_i{PT}_{10i}/{a}_1\kern0.5em and\kern0.5em \overline{PT_{01}}\ {\sum}_i{PT}_{01i}/{a}_0$$

where *PT*_10*i*_ is the number of unexposed control periods (person-time) of case i who is exposed at the case period, *PT*_01*i*_ is the number of exposed control periods (person-time) of case i who is unexposed at the case period, and *a*_1_ is the number of discordant exposed cases with at least one unexposed control period and *a*_0_ is the number of discordant unexposed cases with at least one exposed control period. When ${\sum}_{m=1}^M\mathrm{P}\left(\ {X}_m=0|{X}_0=1\right)$ and ${\sum}_{m=1}^M\mathrm{P}\left(\ {X}_m=1|{X}_0=0\right)$ in Eq. (7) are substituted by $\overline{PT_{10}}$ and $\overline{PT_{01}}$, respectively, we obtain:

9 $${\pi}_{10}={\pi}_1/{\pi}_0={\sum}_{m=1}^M\mathrm{P}\left(\ {X}_m=1|{X}_0=0\right)/{\sum}_{m=1}^M\mathrm{P}\left(\ {X}_m=0|{X}_0=1\right)=\overline{PT_{01}}/\overline{PT_{10}}$$

When standard statistical software is used for conditional logistic regression analysis of case-crossover studies, we introduce weighting to ensure the denominator in Eq. (2) equals that in Eq. (4). Every exposed and unexposed period in case i should be weighted by w_*i*1_ and by w_*i*0_, respectively, defined as follows:

10 $${\mathrm{w}}_{i1}={\pi}_{10}/{m}_i^1\ and\ {\mathrm{w}}_{i0}=1/{m}_i^0\kern0.75em$$

where ${m}_i^1$ is the number of exposed periods and ${m}_i^0$ is the number of the unexposed periods (including both case and control periods) in case i and *π*_10_ is estimated from Eq. (9). In most statistical software, w_*ik*_ (k = 0,1) may be specified as an offset variable in conditional logistic regression which uses the following likelihood:

11 $$L=\prod_{i=1}^N\frac{\exp \left(\beta {x}_{i0}\right)}{\sum_{j=0}^M{\mathrm{w}}_{ij}\exp \left(\beta {x}_{ij}\right)}$$

where w_*ij*_ = w_*i*1_ and w_*ij*_ = w_*i*0_ when j-th period is exposed and unexposed, respectively, in case i.

Using *a*_1_ and *a*_0_, Eq. (4) can be rewritten as

12 $$\mathrm{L}={\left(\frac{\exp \left(\beta \right){\pi}_{10}}{1+{\exp}\left(\beta \right){\pi}_{10}}\right)}^{a_1}{\left(\frac{1}{1+{\exp}\left(\beta \right){\pi}_{10}}\right)}^{a_0}$$

Equation (12) gives (see Additional File 1, Appendix 2) the following maximum likelihood estimate for *OR*_*G*_:

13 $${OR}_G=\exp \left(\beta \right)=\frac{a_1}{a_0}\frac{1}{\pi_{10}}$$

When *π*_10_ estimated by Eq. (9) is considered as a constant, the variance is given by:

14 $$\mathrm{v}\left(\upbeta \right)=\frac{1}{a_0}+\frac{1}{a_1}$$

In order to allow for the variance of the weights estimated by Eq. (9), we recommend the use of bootstrapping to estimate the 95% confidence interval using the 2.5 to 97.5 percentiles of *OR*_*G*_.

From Eqs. (8), (9) and (13) we obtain:

15 $${OR}_G=\exp \left(\upbeta \right)=\frac{a_1}{a_0}\frac{1}{\pi_{10}}=\frac{a_1\ \overline{PT_{10}}}{a_0\ \overline{PT_{01}}}=\frac{\sum_i{PT}_{10i}}{\sum_i{PT}_{01i}}={OR}_{MH}$$

Equation (15) shows that when the model involves only one binary exposure variable, the point estimate of *OR*_*G*_ is the same as *OR*_*MH*_.

#### Case-crossover studies with a binary exposure and a binary time-varying confounder

The Mantel-Haenszel estimator is unbiased for binary exposures when there is pairwise exchangeability. When there is a binary time varying confounder (z), Maclure in his original proposal of the case-crossover design recommended further stratification when using the Mantel-Haenszel method [9]. In a simulation, we followed this recommendation and stratified each subject by z. As a result, control periods where z = 0 were excluded from subjects with z = 1 at the case period. Similarly, control periods where z = 1 were excluded from those with z = 0 at the case period. We have shown that excluding these control periods can lead to bias (see Tables 3 and 4 in Results section).

On the other hand, our method can be extended to studies with time-varying confounder to analyze data of all case and control periods and all periods can be included in the analysis. For example, when there is a binary exposure (x) and a binary time-varying confounder variable (z), *βx*_*ij*_ in the likelihood in Eq. (11) is specified as (*β*, *γ*)(*x*_*ij*_, *z*_*ij*_)^*T*^, which is equal to 0 when (*x*_*ij*_, *z*_*ij*_) =(0,0), *β* when (*x*_*ij*_, *z*_*ij*_) =(1,0), *γ* when (*x*_*ij*_, *z*_*ij*_) =(0,1), and *β* + *γ* when (*x*_*ij*_, *z*_*ij*_) = (1,1) where exp(*γ*) is an estimate of the odds ratio of z. Similarly to the finding that *OR*_*SCL*_ in Eq. (2) is unbiased when within-subject exposure dependency does not exist (Appendix 1, Additional File 1), we may estimate an unbiased *OR*_*G*_ in the model involving the exposure and time-varying confounder by calculating the weight from the exposure variable x (Eq. 10), if within-subject dependency does not exist for z during exposed periods (where the probability that the confounder is positive is *f*_1_) and during unexposed periods (*f*_0_) and the confounder is adjusted for as in the standard conditional logistic regression. Similarly as for a case-crossover study with a binary exposure, we recommend bootstrapped 2.5 to 97.5 percentiles of *OR*_*G*_ to estimating the 95% CI to allow for the variance of the weights (see Appendix 3, Additional File 1 for the detail; as to the relevant SAS codes, see 4-1 h and 4-2f in Appendix 4, Additional File 1).

### Simulation studies

We simulated data relevant to drug therapy with no time trends and with autocorrelated exposure patterns (within-subject dependency) (Appendix 4, Additional File 1). The simulated data is created based on the following observations (i) drug treatment sometimes has a cyclic pattern which often produces within-subject exposure dependency, (ii) some outcomes (e.g., acute adverse events) tend to occur soon after the treatment is initiated but they may also occur later during the drug therapy, and (iii) some patients stop treatment for various reasons while some patients start treatment. When the rate of stopping treatment is the same as that of starting treatment, the stationarity of the exposure may be maintained in the population as follows. We simulated scenarios with and without time varying confounding.

**Fig. 1:**
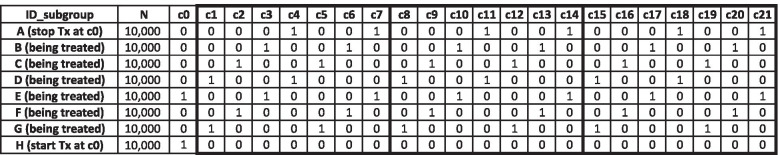
Exposure pattern in a hypothetical dynamic population of 80,000 patients. Patients receiving drug treatment with a cycle of 7 days are divided into 8 Subgroups A to H where 1 period is defined as 1 day. Subgroup A represents stoppers that stop treatment, Subgroup H represents starters that start treatment, and Subgroups B to G represent different exposure patterns of those being treated at the case period. The bolded outline indicates that 21 control periods can be divided into 3 cycles with the same exposure pattern. N: size of each subgroup; c0: exposure status at the case period; cm (m = 1, 2, −--, 21): exposure status at the m-th control period; Tx: treatment

Assume that drug treatment involves a cyclic pattern where one cycle consists of 7 days and a patient has a drug on days 1 and 4 but no drug on days 2, 3, 5, 6, and 7. Figure 1 depicts three cycles of drug treatment with 8 subgroups consisting of 1 case period and 21 control periods, where 1 period is 1 day. Subgroup A represents stoppers who stop treatment at the case period while Subgroup H represents new users who start treatment at the case period. Subgroups B to G represent patients being treated with a different timing relative to the start of the treatment cycle. In Fig. 1, the proportion of those exposed to a drug in the population is always ¼, indicating stationarity (no time trends).

**Fig. 2:**
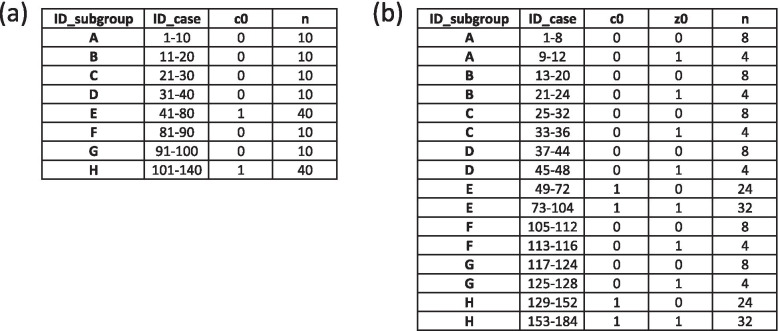
Expected frequency of cases by subgroup in Fig. 1. (**a**) 140 cases who had the outcome at the case period involving one binary exposure variable only, where the incidence rate per period at unexposed period (r0) is assumed to be 0.001 and the rate ratio of the exposure (RR) is assumed to be 4. (**b**) 184 cases who had the outcome at the case period involving one binary exposure variable and one binary time-varying confounder where the incidence rate per period at unexposed period without confounder (r0) is assumed to be 0.001, the rate ratio of the exposure (RR) is assumed to be 4, and that of the time-varying confounder (RRz) is assumed to be 2. The proportion of the time-varying confounder at unexposed periods (f0) is 0.2 and that at exposed periods (f1) in the population 0.4. The status of the confounder at control periods is randomly assigned . n: frequency of cases belonging to each ID_Subgroup in Fig. 1; c0: exposure status at the case period; z0: status of time-varying confounder at the case period

Figure 2a shows 140 cases who had the outcome at the case period when the size of each of Subgroups A to H, *N* = 10,000, the event rate in an unexposed period (*r*_0_) is 0.001 per period and the rate ratio is 4. We assume that the expected number of cases is determined by exposure at the case period only, or *Nr*_0_ when unexposed and *N RR r*_0_ when exposed at the case period.

Figure 2b shows 184 cases who had the outcome at the case period when N = 10,000, the event rate in the unexposed period without time-varying confounding is 0.001 per period and the rate ratio is 4 and 2 for the exposure and time-varying confounder, respectively. The status of the time-varying confounder in the unexposed or exposed period is related to the exposure status of that period only, and *f*_0_ = 0.2 and *f*_1_ = 0.4 where *f*_0_ and *f*_1_ are the expected values of the proportion of exposed periods and unexposed periods in the population, respectively, when the confounder is positive (See Appendix 3, Additional File 1). We assume that the number of cases is as expected, or *Nr*_0_(1 − *f*_0_), *N RR*_*z*_ *r*_0_*f*_0_, *N RR r*_0_(1 − *f*_1_), and *N RR RR*_*z*_ *r*_0_*f*_1_, when the combination of the exposure and time-varying confounder variables (x, z) at the case period is (0, 0), (0, 1), (1, 0), and (1, 1), respectively. The status of the time-varying confounder in the control periods was randomly generated and is therefore independent of the exposure or time-varying confounder at different periods.

To determine the effect of the length of each time period on the estimated odds ratio, we also analyzed the data by dividing 22 days in Fig. 1 into 11, 7, 5, 3, and 2 periods (M = 10, 6, 4, 2, and 1) where 1 period included 2, 3, 4, 7 and 11 days, respectively. The status of the exposure and time varying confounder was defined by the last day of each period (Definition I in Table 1). We analyzed the data by standard conditional regression, the Vines and Farrington method, Mantel-Haenszel methods, and our modified Greenland’s method for the fixed study period of 22 days. With time-varying confounder (z), case and control periods of each subject were further stratified by z in the Mantel-Haenszel method. We used bootstrapping to estimate 2.5 to 97.5 percentiles for *OR*_*G*_. Data simulation and analyses were performed using SAS 9.4 and SAS codes including those for bootstrap method are shown in Appendix 4 in Additional File 1.

**Table 1:** Exposure Definitions

	Case period	Control periods
Definition I	Last day	Last day
Definition II	Last day	Half or more days
Definition III	Last day	Any 1 day
Definition IV	Any 1 day	Any 1 day

Last day = exposure status of the period was the exposure status on the last day; Half or more days = exposure status of the period is defined as ‘exposed’ if at least half of days during the period was exposed and ‘unexposed’ otherwise; Any 1 day = exposure status of the period is defined as ‘exposed’ if at least 1 day during the period is exposed and ‘unexposed’ otherwise

### Case-crossover studies of real-world data

The method was also applied to data from Japanese and Australian databases. The Japanese study on the association between celecoxib and peripheral edema from a previous study [22] was approved by the ethics committee of Tokyo University of Science (approval number: 18023) where obtaining the informed consent from study subjects was waived for the current study. The Japanese data came from 25 corporate-type health insurance plans extracted from Cross-Fact database [23]. Claims data covering 60 months between May 2013 to April 2018 included 1,163,968 males (age (SD) = 42.5 (13.2) years old) and 1,349,901 females (42.2 (13.1) years old) who were 20 years old or older (but younger than 75 years old). As detailed in Additional File 1 (Appendix 5), we examined 99,821 new users of celecoxib, who used celecoxib after at least 180 days of non-use. The occurrence of peripheral edema was defined by new use of furosemide after at least 180 days of non-use and the index date was the day when the outcome occurred. Daily exposure during an 84-day study period was determined using a 7-day grace period. We selected 311 cases who had both exposed and unexposed days during the study period. The 84-day study period was divided into (M + 1) periods where M = 1, 2, 5, 11, 27 and 83, resulting in periods of 42, 28, 14, 7, 3 and 1 days, respectively. Four different definitions were used to determine exposure in the case and control periods as shown in Table 1. Cases who started celecoxib on the index date were excluded from the analysis since furosemide could have been prescribed for prevention rather than treatment of edema.

The data was analyzed by standard conditional logistic regression (Eq. (2)) and the Mantel-Haenszel method with their 95% CIs, and the weighting method for a binary exposure (Eq. (11)) with 2.5 to 97.5 percentiles estimated by the bootstrap method. We also extended the study period to 168 and 336 days. All the analyses were performed using SAS 9.4. Data and SAS codes to analyze the data are available upon request.

The data for the Australian study on the association between hip fracture and psychoactive medicines [24, 25] were obtained from the Australian Department of Veterans’ Affairs administrative claims database. The study was approved by Department of Defense and Veterans’ Affairs Human Research Ethics (E016–007) and University of South Australia Human Research Ethics (P203/04) where obtaining the informed consent from study subjects was waived for the current study. The cases were 8828 patients aged over 65 years who were hospitalized for hip fracture between 2009 and 2012. The index date for each case was the date of hospitalization. We have re-analyzed the data from the previous case-control study [25] as a case-crossover study with SSRIs as the exposure using the same methods as the Japanese study.

Both of the studies in Japan and Australia were carried out in accordance with the Declaration of Helsinki, and all methods were carried out in accordance with relevant guidelines and regulations in Japan and Australia.

## Results

### Simulation studies: comparison of methods with or without time-varing confounding

**Fig. 3:**
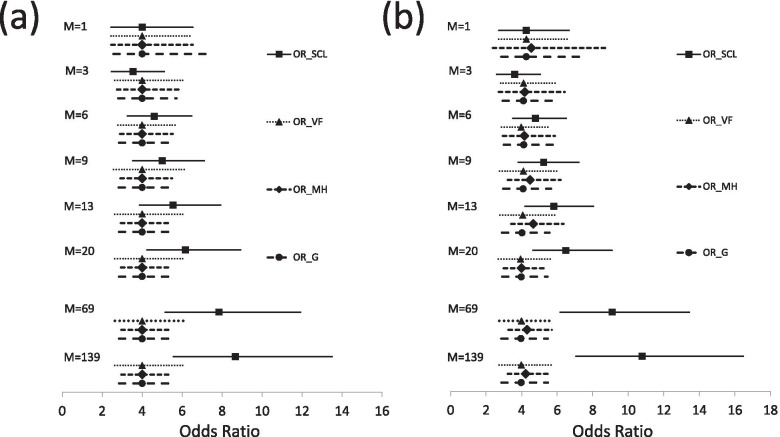
Estimates of OR_SCL, OR_VF, OR_MH and OR_G for simulated data in Fig. 1**.** OR_SCL, OR_VF and OR_MH with their 95% CI, and OR_G with its 2.5–97.5pct for the exposure (exp(*β*)) for M = 1, 3, 6, 9, 13, 20, 69, and 139, where M is the number of control periods and study period = M + 1 days (1 period = 1 day). (**a**) shows results for the population in Fig. 2a with one binary exposure variable only. (**b**) shows results for the population in Fig. 2b with one binary exposure variable and one binary time-varying confounder. OR_SCL: odds ratio by the standard conditional logistic regression (*OR*_*SCL*_); OR_VF: odds ratio by the Vines and Farrington’s method (*OR*_*VF*_); OR_MH: odds ratio by the Mantel-Haenszel method (*OR*_*MH*_); OR_G: odds ratio by the Greenland’s method (*OR*_*G*_); 95%CI: 95% confidence interval; 2.5–97.5pct: 2.5 to 97.5 percentiles

Table 2 and Fig. 3a show *OR*_*SCL*_, *OR*_*VF*_, and *OR*_*MH*_ with their 95% CIs, and *OR*_*G*_ with its 2.5 to 97.5 percentiles estimated for the scenario in Fig. 2a for M control periods (1 period = 1 day). The difference between *OR*_*SCL*_ and true RR (4.0) was more than 10% of the true value when M = 6 or > 7 and increased when M increased. When control periods were extended to 10 and 20 cycles (69 and 139 control periods), the estimate (95% CI) of *OR*_*SCL*_ was 7.92 (5.22–12.03) and 8.71 (5.59–13.57), respectively (not shown in Table 2). On the other hand, odds ratios from the remaining 3 methods (*OR*_*VF*_, *OR*_*MH*_, and *OR*_*G*_) were unbiased irrespective of the value of M. The estimate of *OR*_*VF*_ cannot be estimated when M = 7, 10, 14, 17 and 21 because the positivity condition was not satisfied for any data. For example, when M = 7, ∑*P*(*X*_*i*0_ = 0, *N*_*exposed*_ = 1) = 0 because *X*_*i*0_ = 1 in Subgroup H which is only one subgroup where *N*_*exposed*_ = 1 and similarly for *N*_*exposed*_ = 2 or 3, *X*_*i*0_ was the same for all subgroups.

**Table 2:** The estimates of *OR*_*SCL*_, *OR*_*VF*_, *OR*_*MH*_, and *OR*_*G*_ from simulated data with a binary exposure only (study period = M + 1 days). Results are shown graphically in Fig. 3a

M	*OR*_*SCL*_ (95%CI)	*OR*_*VF*_ (95%CI)	*OR*_*MH*_ (95%CI)	*OR*_*G*_ (2.5–97.5pct)
1	4.00 (2.45–6.53)	4.00 (2.45–6.53)	4.00 (2.45–6.53)	4.00 (2.57–7.33)
2	4.00 (2.74–5.85)	4.00 (2.74–5.85)	4.00 (2.74–5.85)	4.00 (2.80–6.00)
3	3.55 (2.47–5.10)	4.00 (2.64–6.06)	4.00 (2.73–5.86)	4.00 (2.80–5.72)
4	3.84 (2.72–5.41)	4.00 (2.80–5.71)	4.00 (2.81–5.69)	4.00 (2.82–5.64)
5	4.28 (3.04–6.03)	4.00 (2.86–5.59)	4.00 (2.85–5.61)	4.00 (2.84–5.73)
6	4.60 (3.27–6.48)	4.00 (2.80–5.71)	4.00 (2.89–5.53)	4.00 (2.84–5.70)
7	4.38 (3.10–6.20)	–	4.00 (2.87–5.57)	4.00 (2.85–5.66)
8	4.73 (3.34–6.70)	4.00 (2.34–6.84)	4.00 (2.89–5.54)	4.00 (2.88–5.63)
9	5.01 (3.53–7.10)	4.00 (2.58–6.20)	4.00 (2.91–5.50)	4.00 (2.83–5.65)
10	4.86 (3.41–6.94)	–	4.00 (2.89–5.54)	4.00 (2.84–5.70)
11	5.09 (3.57–7.26)	4.00 (2.00–8.00)	4.00 (2.91–5.51)	4.00 (2.84–5.64
12	5.33 (3.73–7.62)	4.00 (2.49–6.42)	4.00 (2.92–5.48)	4.00 (2.85–5.60)
13	5.54 (3.88–7.92)	4.00 (2.64–6.06)	4.00 (2.93–5.46)	4.00 (2.85–5.67)
14	5.39 (3.75–7.73)	–	4.00 (2.92–5.49)	4.00 (2.85–5.66)
15	5.60 (3.90–8.05)	4.00 (2.34–6.84)	4.00 (2.92–5.47)	4.00 (2.87–5.61)
16	5.79 (4.02–8.33)	4.00 (2.58–6.20)	4.00 (2.93–5.46)	4.00 (2.84–5.60)
17	5.66 (3.92–8.18)	–	4.00 (2.92–5.48)	4.00 (2.84–5.65)
18	5.83 (4.03–8.43)	4.00 (2.00–8.00)	4.00 (2.93–5.46)	4.00 (2.84–5.64)
19	6.00 (4.14–8.69)	4.00 (2.49–6.42)	4.00 (2.93–5.45)	4.00 (2.84–5.61)
20	6.16 (4.25–8.92)	4.00 (2.64–6.06)	4.00 (2.94–5.44)	4.00 (2.85–5.65)
21	6.02 (4.14–8.75)	–	4.00 (2.93–5.46)	4.00 (2.85–5.66)

M is the number of control periods and study period = (M + 1) days. 1 period = 1 day for all values of M

*OR*_*SCL*_: odds ratio by the standard conditional logistic regression; *OR*_*VF*_: odds ratio by the Vines and Farrington’s method; *OR*_*MH*_: odds ratio by the Mantel-Haenszel method; *OR*_*G*_: odds ratio by the Greenland’s method; 95%CI: 95% confidence interval; 2.5–97.5pct: 2.5 to 97.5 percentiles

Table 3 and Appendix Fig. 3b show *OR*_*SCL*_, *OR*_*VF*_, and *OR*_*MH*_ with their 95% CIs, and *OR*_*G*_ with its 2.5 to 97.5 percentiles estimated for the time varying confounding scenario in Fig. 2b for M control periods (1 period = 1 day). The difference between *OR*_*SCL*_ for exp(*β*) and the true RR (4.0) was more than 10% of the true value when M > 5 and increased when M increased. The *OR*_*SCL*_ estimate for exp(*β*) was larger than the corresponding value in Table 2. When control periods were extended to 10 and 20 cycles, the point estimate of *OR*_*SCL*_ for exp(*β*) was 9.18 (6.23–13.52) and 10.84 (7.09–16.56), respectively. On the other hand, *OR*_*VF*_ and *OR*_*G*_ for both exp(*β*) and exp(*γ*) were in general unbiased, particularly when M > 7 where the estimates of *OR*_*VF*_ and *OR*_*G*_ for exp(*β*) were within 3% of the true value. The estimate of *OR*_*MH*_ for exp(*β*) varied between 3.82 (M = 2) and 4.65 (M = 13). Though not shown in Table 3 and Fig. 3bb, *OR*_*MH*_ for exp(*β*) estimated by ignoring the time-varying confounding was stable but overestimated as 4.67 (17% above the true value). When M = 1, *OR*_*VF*_ was 4.27 for exp(*β*) (about 7% overestimated) and 1.86 for exp(*γ*) (7% underestimated). Similarly, when M = 1, *OR*_*G*_ was 4.26 for exp(*β*) (7% overestimated) and 1.68 for exp(*γ*) (16% underestimated). When N was increased to 1,000,000, exp(*β*) and *e*xp(*γ*) were 3.96 and 2.09 for *OR*_*VF*_ and 3.97 and 2.04 for *OR*_*G*_ when M = 1 (not shown in Table 3).

**Table 3:** Estimates of *OR*_*SCL*_, *OR*_*VF*_, *OR*_*MH*_, and *OR*_*G*_ from simulated data with a binary exposure and a binary time-varying confounder (study period = M + 1 days). Results are shown graphically in Fig. 3b

M variables		*OR*_*SCL*_ (95%CI)	*OR*_*VF*_ (95%CI)	*OR*_*MH*_ (95%CI)	*OR*_*G*_ (2.5–97.5pct)
1	exp(*β*)	4.26 (2.72–6.66)	4.27 (2.73–6.68)	4.55 (2.37–8.73)	4.26 (2.85–7.33)
	exp(*γ*)	1.68 (1.02–2.78)	1.86 (1.11–3.11)		1.68 (1.03–2.96)
2	exp(*β*)	4.04 (2.85–5.75)	4.04 (2.85–5.73)	3.82 (2.49–5.86)	4.04 (2.78–6.11)
	exp(*γ*)	1.84 (1.15–2.94)	2.06 (1.28–3.30)		1.84 (1.19–2.92)
3	exp(*β*)	3.62 (2.60–5.04)	4.11 (2.83–5.97)	4.17 (2.71–6.43)	4.10 (2.93–5.82)
	exp(*γ*)	2.12 (1.43–3.12)	1.93 (1.31–2.86)		2.21 (1.45–3.37)
4	exp(*β*)	3.91 (2.86–5.34)	4.19 (3.03–5.79)	3.91 (2.66–5.75)	4.09 (3.00–5.67)
	exp(*γ*)	2.14 (1.48–3.11)	2.43 (1.66–3.54)		2.20 (1.45–3.26)
5	exp(*β*)	4.36 (3.18–5.97)	4.07 (3.00–5.51)	4.04 (2.78–5.86)	4.02 (2.93–5.57)
	exp(*γ*)	1.86 (1.29–2.66)	2.35 (1.62–3.41)		1.92 (1.28–2.86)
6	exp(*β*)	4.78 (3.51–6.52)	3.98 (2.88–5.50)	4.15 (2.93–5.88)	4.12 (3.00–5.77)
	exp(*γ*)	1.92 (1.36–2.71)	2.00 (1.37–2.92)		1.87 (1.33–2.63)
7	exp(*β*)	4.48 (3.26–6.15)	–	4.42 (3.10–6.30)	4.04 (2.94–5.59)
	exp(*γ*)	1.89 (1.34–2.67)	2.03 (1.33–3.08)		1.86 (1.30–2.70)
8	exp(*β*)	4.90 (3.57–6.74)	4.07 (2.50–6.63)	3.88 (2.79–5.42)	4.07 (2.93–5.68)
	exp(*γ*)	1.81 (1.29–2.53)	1.76 (1.17–2.64)		1.79 (1.24–2.51)
9	exp(*β*)	5.24 (3.81–7.22)	4.10 (2.76–6.08)	4.47 (3.22–6.19)	4.08 (2.96–5.65)
	exp(*γ*)	1.77 (1.27–2.48)	2.10 (1.42–3.10)		1.75 (1.23–2.48)
10	exp(*β*)	5.08 (3.67–7.03)	–	4.13 (2.99–5.70)	4.06 (2.94–5.61)
	exp(*γ*)	1.77 (1.28–2.45)	1.62 (1.08–2.42)		1.76 (1.26–2.50)
11	exp(*β*)	5.33 (3.85–7.38)	4.07 (2.17–7.64)	4.15 (3.01–5.72)	4.07 (2.96–5.65)
	exp(*γ*)	1.78 (1.29–2.46)	1.86 (1.26–2.76)		1.73 (1.23–2.38)
12	exp(*β*)	5.61 (4.06–7.75)	4.12 (2.69–6.32)	4.51 (3.29–6.18)	4.07 (2.95–5.62)
	exp(*γ*)	1.92 (1.39–2.66)	1.81 (1.24–2.64)		1.86 (1.34–2.62)
13	exp(*β*)	5.81 (4.19–8.04)	4.06 (2.79–5.91)	4.65 (3.40–6.36)	4.02 (2.90–5.56)
	exp(*γ*)	1.93 (1.39–2.68)	1.87 (1.28–2.73)		1.88 (1.34–2.61)
14	exp(*β*)	5.60 (4.02–7.79)	–	4.05 (2.96–5.55)	3.99 (2.87–5.53)
	exp(*γ*)	1.93 (1.40–2.66)	2.10 (1.40–3.13)		1.87 (1.35–2.57)
15	exp(*β*)	5.83 (4.17–8.13)	3.94 (2.42–6.42)	3.85 (2.85–5.21)	3.97 (2.86–5.50)
	exp(*γ*)	1.91 (1.38–2.63)	2.04 (1.39–3.00)		1.86 (1.35–2.57)
16	exp(*β*)	6.05 (4.35–8.43)	3.98 (2.68–5.92)	3.88 (2.87–5.25)	3.98 (2.88–5.52)
	exp(*γ*)	2.04 (1.47–2.82)	2.11 (1.45–3.07)		1.98 (1.41–2.72)
17	exp(*β*)	5.94 (4.25–8.30)	–	4.21 (3.09–5.73)	3.97 (2.87–5.51)
	exp(*γ*)	2.06 (1.49–2.84)	2.19 (1.48–3.23)		2.02 (1.46–2.79)
18	exp(*β*)	6.14 (4.39–8.59)	4.08 (2.18–7.64)	4.13 (3.05–5.59)	3.99 (2.89–5.51)
	exp(*γ*)	2.02 (1.47–2.77)	2.16 (1.48–3.15)		1.95 (1.43–2.65)
19	exp(*β*)	6.32 (4.52–8.85)	4.00 (2.60–6.14)	3.94 (2.94–5.29)	3.98 (2.87–5.48)
	exp(*γ*)	2.07 (1.51–2.85)	1.98 (1.37–2.88)		2.01 (1.50–2.71)
20	exp(*β*)	6.49 (4.64–9.08)	3.94 (2.71–5.74)	3.99 (2.98–5.34)	3.98 (2.90–5.46)
	exp(*γ*)	2.16 (1.57–2.98)	2.14 (1.47–3.11)		2.05 (1.52–2.76)
21	exp(*β*)	6.35 (4.52–8.91)	–	4.16 (3.09–5.61)	3.96 (2.88–5.46)
	exp(*γ*)	2.20 (1.60–3.01)	2.20 (1.51–3.22)		2.10 (1.53–2.81)

M is the number of control periods and study period = (M + 1) days. 1 period = 1 day for all values of M

*OR*_*SCL*_: odds ratio by the standard conditional logistic regression; *OR*_*VF*_: odds ratio by the Vines and Farrington’s method; *OR*_*MH*_: odds ratio by the Mantel-Haenszel method; *OR*_*G*_: odds ratio by the Greenland’s method; 95%CI: 95% confidence interval; 2.5–97.5pct: 2.5 to 97.5 percentiles; exp(*β*): estimate for the exposure variable; exp(*γ*): estimate for the time-varying confounder

**Fig. 4:**
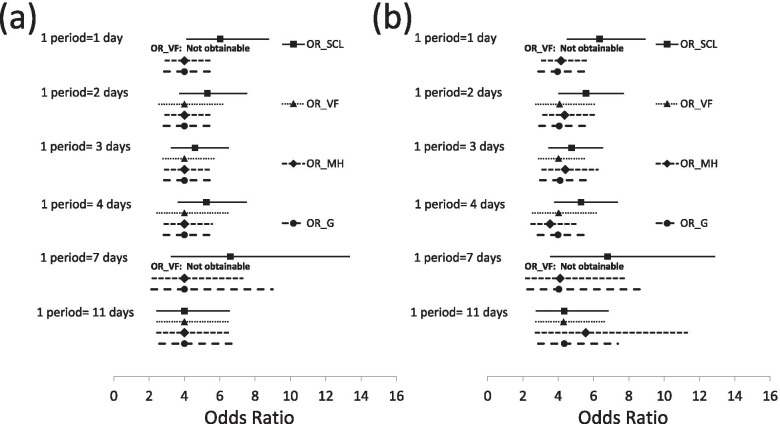
Estimates of OR_SCL, OR_VF, OR_MH and OR_G for simulated data with a fixed study period. OR_SCL, OR_VF and OR_MH with their 95% CI, and OR_G with its 2.5–97.5pct for the exposure (exp(*β*)) where study period is fixed as 22 days but the number of days in 1 period is varied. When 1 period = 1 or 7 days, OR_VF is not obtainable because the positivity condition is not satisfied. (**a**) one binary exposure variable only. (**b**) one binary exposure variable and one binary time-varying confounder. OR_SCL: odds ratio by the standard conditional logistic regression (*OR*_*SCL*_); OR_VF: odds ratio by the Vines and Farrington’s method (*OR*_*VF*_); OR_MH: odds ratio by the Mantel-Haenszel method (*OR*_*MH*_); OR_G: odds ratio by the Greenland’s method (*OR*_*G*_)

Table 4 and Fig. 4a and b show *OR*_*SCL*_, *OR*_*VF*_, and *OR*_*MH*_ with their 95% CIs, and *OR*_*G*_ with its 2.5 to 97.5 percentiles for the scenarios in Fig. 2a and b, where the length of the study period was fixed as 22 days but the length of each time period varied between 1 and 11 days. For the scenario in Fig. 2a with one binary exposure variable only, *OR*_*SCL* _ for exp(*β*) varied between 4.00 and 6.61 when the length of the time period varied, but *OR*_*VF*_, *OR*_*MH*_, and *OR*_*G*_ were stable and unbiased (see Table 4 and Fig. 4a). For the scenario in Fig. 2b with one binary exposure variable and one time varying confounder randomly generated in the control periods, the *OR*_*SCL*_ estimate for exp(*β*) varied between 4.34 (1 period = 11 days) and 6.79 (1 period = 7 days) when M and the length of the time period varied, while *OR*_*VF*_ and *OR*_*G*_ for exp(*β*) were stable and close to the true value, though the odds ratio was a little overestimated as 4.34 when the time period was 11 days (see Table 4 and Fig. 4b). On the other hand, *OR*_*MH*_ for exp(*β*) varied 3.54 (1 period = 4 days) and 5.56 (1 period = 11 days). Though not shown in Table 4 and Fig. 4b, *OR*_*MH*_ for exp(*β*) estimated by ignoring the time-varying confounding was stable but overestimated as 4.67. When M = 1 (1 period = 11 days), *OR*_*VF*_ was 4.31 (8% overestimated) for exp(*β*) and 1.94 (3% underestimated) for exp(*γ*). Similarly, when M = 1, *OR*_*G*_ was 4.34 (9% overestimated) for exp(*β*) and 1.35 (32% underestimated) for exp(*γ*). When N was increased to 1,000,000, exp(*β*) and *e*xp(*γ*) were 3.97 and 1.95 for both *OR*_*VF*_ and *OR*_*G*_ when M = 1 (not shown in Table 4).

**Table 4:** Estimates of *OR*_*SCL*_, *OR*_*VF*_ and *OR*_*MH*_ (95% CI), and *OR*_*MH*_ (2.5–97.5pct): Simulated data (study period = 22 days). Results are shown graphically in Fig. 4

Days in 1 period	1 day	2 days	3 days	4 days	7 days	11 days
M	21	10	6	4	2	1
One binary exposure variable only						
*OR*_*SCL*_ exp(*β*)	6.02 (4.14–8.75)	5.30 (3.73–7.53)	4.60 (3.27–6.48)	5.23 (3.65–7.50)	6.61 (3.27–13.33)	4.00 (2.45–6.53)
*OR*_*VF*_ exp(*β*)	–	4.00 (2.58–6.20)	4.00 (2.80–5.71)	4.00 (2.45–6.53)	–	4.00 (2.45–6.53)
*OR*_*MH*_ exp(*β*)	4.00 (2.93–5.46)	4.00 (2.92–5.48)	4.00 (2.89–5.53)	4.00 (2.87–5.57)	4.00 (2.19–7.29)	4.00 (2.45–6.53)
*OR*_*G*_ exp(*β*)	4.00 (2.85–5.66)	4.00 (2.82–5.60)	4.00 (2.84–5.70)	4.00 (2.82–5.81)	4.00 (2.12–9.00)	4.00 (2.57–6.69)
One binary exposure variable and one binary time-varying confounder						
*OR*_*SCL*_ exp(*β*)	6.35 (4.52–8.91)	5.57 (4.04–7.68)	4.76 (3.49–6.51)	5.29 (3.81–7.35)	6.79 (3.59–12.86)	4.34 (2.76–6.82)
exp(*γ*)	2.20 (1.60–3.01)	1.78 (1.28–2.48)	1.80 (1.27–2.54)	2.06 (1.39–3.06)	1.91 (1.25–2.91)	1.35 (0.83–2.20)
*OR*_*VF*_ exp(*β*)	–	4.08 (2.74–6.07)	4.02 (2.90–5.58)	4.02 (2.57–6.29)	–	4.31 (2.75–6.74)
exp(*γ*)	2.20 (1.51–3.22)	1.78 (1.20–2.63)	1.83 (1.25–2.67)	2.02 (1.24–3.31)	2.34 (1.43–3.85)	1.94 (1.16–3.24)
*OR*_*MH*_ exp(*β*)	4.16 (3.09–5.61)	4.36 (3.15–6.03)	4.40 (3.11–6.24)	3.54 (2.49–5.04)	4.11 (2.17–7.80)	5.56 (2.73–11.30)
*OR*_*G*_ exp(*β*)	3.96 (2.88–5.46)	4.06 (2.93–5.61)	4.11 (2.97–5.78)	4.00 (2.85–5.67)	4.04 (2.26–8.80)	4.34 (2.86–7.40)
exp(*γ*)	2.10 (1.53–2.81)	1.77 (1.25–2.47)	1.76 (1.23–2.53)	2.01 (1.39–2.83)	1.90 (1.27–2.86)	1.35 (0.80–2.26)

M is the number of control periods while study period is fixed as 22 days (precisely, study period = Int(22/(M + 1)) *(M + 1)) days)

*OR*_*SCL*_: odds ratio by the standard conditional logistic regression; *OR*_*VF*_: odds ratio by the Vines and Farrington’s method; *OR*_*MH*_: odds ratio by the Mantel-Haenszel method; *OR*_*G*_: odds ratio by the Greenland’s method; 95%CI: 95% confidence interval; 2.5–97.5pct: 2.5 to 97.5 percentiles; exp(*β*): the estimate for the rate ratio for the exposure; exp(*γ*): the estimate for the rate ratio for the time-varying confounder

### Analyses of case-crossover studies of real-world data

Tables 5 and 6 show the estimates using Exposure Definition I in Table 1 in the Japanese study. In general, *OR*_*SCL*_ was different from *OR*_*G*_ and *OR*_*MH*_ except when M = 1, and *OR*_*SCL*_ estimated from Eq. (2) increased when study period increased: *OR*_*SCL*_ was between 1.91 and 2.82 when study period = 84 days (Table 5), between 2.13 and 4.19 when study period = 168 days (Table 6), and between 3.73 and 7.16 when study period = 336 days (Table 6). The point estimate of *OR*_*G*_ from Eq. (4) was always the same as that of *OR*_*MH*_, as expected.

**Table 5:** Estimates of *OR*_*SCL*_ and *OR*_*MH*_ (95% CI), *OR*_*G*_ (2.5–97.5pct): Japanese data on celecoxib-peripheral edema with study period = 84 days and Exposure Definition I

Study period	84 days					
Days in 1 period	1 day	3 days	7 days	14 days	28 days	42 days
M	83	27	11	5	2	1
*OR*_*SCL*_	2.82 (2.14–3.71)	2.73 (2.08–3.60)	2.53 (1.93–3.33)	2.28 (1.74–2.99)	2.05 (1.54–2.74)	1.91 (1.38–2.64)
*OR*_*MH*_	1.99 (1.58–2.51)	1.98 (1.57–2.50)	1.97 (1.55–2.50)	1.94 (1.51–2.48)	1.93 (1.46–2.56)	1.91 (1.38–2.64)
*OR*_*G*_	1.99 (1.49–2.65)	1.98 (1.48–2.64)	1.97 (1.48–2.62)	1.94 (1.44–2.58)	1.93 (1.43–2.62)	1.91 (1.36–2.72)

M is the number of control periods

*OR*_*SCL*_: odds ratio by the standard conditional logistic regression; *OR*_*MH*_: odds ratio by the Mantel-Haenszel method; *OR*_*G*_: odds ratio by the Greenland’s method; 95%CI: 95% confidence interval; 2.5–97.5pct: 2.5 to 97.5 percentiles

**Table 6:** Estimates of *OR*_*SCL*_ and *OR*_*MH*_ (95% CI), and *OR*_*G*_ (2.5–97.5pct): Japanese data on celecoxib-peripheral edema with study period = 168 and 336 days and Exposure Definition I

Study period	168 days			336 days		
Days in 1 period	1 day	14 days	84 days	1 days	14 days	168 days
M	167	11	1	335	23	1
*OR*_*SCL*_	4.19 (3.16–5.55)	3.39 (2.58–4.45)	2.13 (1.54–2.95)	7.16 (5.27–9.74)	5.97 (4.43–8.05)	3.73 (2.42–5.75)
*OR*_*MH*_	2.45 (1.94–3.09)	2.38 (1.87–3.03)	2.13 (1.54–2.95)	2.95 (2.28–3.82)	2.92 (2.24–3.79)	3.73 (2.42–5.75)
*OR*_*G*_	2.45 (1.87–3.25)	2.38 (1.82–3.15)	2.13 (1.57–2.99)	2.95 (2.14–4.09)	2.92 (2.12–4.03)	3.73 (2.49–6.06)

M is the number of control periods

*OR*_*SCL*_: odds ratio by the standard conditional logistic regression; *OR*_*MH*_: odds ratio by the Mantel-Haenszel method; *OR*_*G*_: odds ratio by the Greenland’s method. 95%CI: 95% confidence interval; 2.5–97.5pct: 2.5 to 97.5 percentiles

In Appendix Tables 5a and 5b in Additional File 1, the results corresponding to Table 5 and Table 6 for Exposure Definitions II, III and IV (Table 1) are presented. Those results indicated, as in Tables 5 and 6, that *OR*_*SCL*_ varied when M varied as well as when the study period varied, while *OR*_*MH*_ and *OR*_*G*_ were relatively stable. Detailed description for Definition II, III and IV is given in Appendix 5 in Additional File 1.

In the Australian study, after excluding the concordant cases, there were 1316 discordant cases with daily exposed and unexposed periods to SSRIs in the 180 days before the index date. Exposure on the index date was excluded since hip fracture may have occurred the day before admission to hospital.

We used periods of 1, 5, 20, 30, 60 and 90 days with M = 179, 35, 8, 5, 2 and 1, respectively. Exposure within each period was defined using Definition I (Table 1). Estimates of the *OR*_*SCL*_ were biased upwards (Table 7). When M = 1 (exposure period = 90 days), estimates for *OR*_*SCL*_ was identical to *OR*_*MH*_ and *OR*_*G*_ as expected. As in the Japanese study, *OR*_*SCL*_ from Eq. (2) increased with M for M > 1.

**Table 7:** Estimates of *OR*_*SCL*_ and *OR*_*MH*_ (95% CI), and *OR*_*G*_ (2.5–97.5pct): Australian data on SSRI-hip fracture (Study period = 180 days, Exposure Definition I)

Study period	180 days					
Days in 1 period	1 day	5 days	20 days	30 days	60 days	90 days
M	179	35	8	5	2	1
*OR*_*SCL*_	2.02 (1.71–2.37)	1.85 (1.58–2.17)	1.76 (1.49–2.06)	1.72 (1.46–2.02)	1.59 (1.33–1.91)	1.51 (1.24–1.83)
*OR*_*MH*_	1.67 (1.46–1.92)	1.60 (1.39–1.84)	1.59 (1.37–1.84)	1.61 (1.38–1.88)	1.55 (1.30–1.85)	1.51 (1.24–1.83)
*OR*_*G*_	1.67 (1.49–1.90)	1.60 (1.40–1.83)	1.59 (1.39–1.83)	1.61 (1.41–1.87)	1.55 (1.30–1.81)	1.51 (1.24–1.85)

M is the number of control periods

*OR*_*SCL*_: odds ratio by the standard conditional logistic regression; *OR*_*MH*_: odds ratio by the Mantel-Haenszel method; *OR*_*G*_: odds ratio by the Greenland’s method; 95%CI: 95% confidence interval; 2.5–97.5pct: 2.5 to 97.5 percentiles

## Discussion

### Methods to remove BIAS from within-subject exposure dependency with or without time-varying confonders

Using simulated data, we showed that *OR*_*SCL*_ can be biased when there is within-subject exposure dependency but no exposure time trend, except when only one control period is used. When only one control period is used (M = 1), pairwise exchangeability is equivalent to global exchangeability and bias due to within-subject exposure dependency in standard conditional logistic regression does not occur (although bias due to time trends may occur). In Tables 2 and 3, *OR*_*SCL*_ increased with the increase of study period. This observation is similar to that in the previous case-crossover studies, where the odds ratio increased when a longer study period was employed [27–29]. In Table 2, *OR*_*VF*_, *OR*_*MH*_, and *OR*_*G*_ were unbiased. Of those 3 unbiased estimates, *OR*_*VF*_ may be difficult to calculate when analyzing real-world data because it requires population data (or samples from the population) to estimate the exposure probabilities, unlike *OR*_*MH*_ and *OR*_*G*_. In addition, probabilities for many exposure permutations must be reliably estimated which may be intractable. For example, in Fig. 1 with 21 control periods, only 8 subgroups A to H were assumed to exist, but in real-world data, many more exposure patterns would occur. It is possible that the positivity condition is not satisfied for certain permutations, and these do not contribute to the estimation of *OR*_*VF*_. These limitations make *OR*_*VF*_ difficult to use in practical applications.

As Vines and Farrington showed, when no exposure time trend exists and there is one binary exposure variable which is pairwise exchangeable, *OR*_*MH*_ and *OR*_*G*_ are unbiased even when *OR*_*SCL*_ is biased. However, when the model involves a time-varying confounder in addition to the exposure variable, *OR*_*MH*_ may be biased when periods of each subject are further stratified by the time-varying confounder as in Tables 3 and 4. In contrast, the time-varying confounder can be handled by *OR*_*G*_. Results shown in Tables 3 and 4 indicate that our proposed method is unbiased when both within-subject exposure dependency and time-varying confounding occur at the same time, provided that the time-varying confounder is independent between periods.

In Tables 3 and 4, when M = 1 (i.e., only 1 control period is used), *OR*_*G*_ and *OR*_*VF*_ for exp(*β*) were overestimated and those for exp(*γ*) were underestimated, but the estimates approached to the true values when N was increased, suggesting that the deviation from the true values observed when M = 1 was due to random error. Conversely, it is likely that employing a larger number of control periods can produce more precise point estimates of exp(*β*) and exp(*γ*).

### Real-world data analysis

In the Japanese and Australian studies, we found a discrepancy between *OR*_*SCL*_ and *OR*_*G*_ when more than one control period was used. In the Japanese study, *OR*_*SCL*_ was more than 2 times larger than *OR*_*G*_ when the study period increased. We believe that this discrepancy occurred mainly due to within-subject exposure dependency, which increases as the study period increases. The estimates of *OR*_*G*_ were the same as *OR*_*MH*_ as expected.

In the Australian study, the discrepancy between *OR*_*SCL*_ and *OR*_*G*_ was modest compared with the Japanese study. This was compatible with the finding by Vines and Farrington that the bias due to within-subject exposure dependency is minimal when exp(β) ≈ 1 [15].

### Limitations of the current study and future direction

In the current study, our focus was on bias from within-subject exposure dependency. However, biases can occur from other sources as well. First, we have not applied the weighting method when a time trend in the exposure exists [14]. Though it was shown in the methods section that if there is no time trend, probabilities that the individual is exposed (*π*_1_) and unexposed (*π*_0_) at the case period can be estimated without data from population or time-controls, to check whether assumptions of pairwise exchangeability (no exposure time trend) are satisfied, we need data of the population or time-controls. When the proportion of periods exposed in the population or time-controls is roughly constant over study periods, this will support pairwise exchangeability assumptions. When time trend exists, case-time-control design [14] may be used, but more study is needed when both exposure time trend and within-subject exposure dependency exist. Second, a ‘washout period’ has been used in some case-crossover studies [16, 17, 19] to allow for the uncertainty in the optimal length of one period and to reduce within subject exposure dependency. In the current study, when the length of the time period varied, *OR*_*G*_ (and *OR*_*MH*_) was stable in both the simulation study and real-world data. Since exposure was defined by the last day of the period, any period with 2 or more days was equivalent to using a ‘washout period’ at the beginning of the period. However, much more analyses are needed to examine the need for a ‘washout period’ and to determine the optimal length of one period particularly when within-subject exposure dependency exists. Another bias may occur when the event rate is not constant. For example, the event rate may be particularly high soon after the exposure is started, compared to later after treatment was started. One solution for this problem is to divide the exposed periods into high-risk and low-risk periods and considering this as different levels of exposure. When within-subject exposure dependency exists, this will require weighting for at least 3 exposure levels and the weighting method for binary exposures described in this study should be expanded for multi-level exposures.

Limitations of our study include that we assumed a time-varying confounder with no within-subject dependency. If within-subject dependency exists for a time-varying confounder, the weighting method may need to allow for both the exposure and time-varying confounders. Furthermore, when unmeasured time-varying confounders exist, the results still can be biased even when using our weighting method.

Finally, as mentioned earlier, the variance of *OR*_*G*_ in Eq. (14) is estimated assuming the weight in Eq. (10) is a constant. To allow for uncertainty in estimating the weights in Eq. (10), bootstrapping was used to estimate to 95% CI from 2.5 to 97.5 percentiles of *OR*_*G*_. However, an analytical method to estimate the variance of *OR*_*G*_ which accounts for the variance of the weights may be useful and be developed in a future study.

## Conclusion

Despite autocorrelated exposures being common in pharmacoepidemiology, bias due to within-subject exposure dependency in the case-crossover study has had little attention in the pharmacoepidemiology literature and standard conditional logistic regression has been widely used without assessing whether this bias exists. Although using only one control period can avoid bias due to within-subject exposure dependency, this will reduce the accuracy of the estimate due to random error, as seen in our simulation study (the odds ratio when M = 1 in Tables 3 and 4). To assess for the possibility of bias, we recommend comparing the Mantel-Haenszel odds ratio with the standard conditional logistic regression odds ratio before starting analysis of case-crossover data. If time-varying confounders exist in data set, they may be ignored when comparing two odds ratios to assess the possibility of bias due to within-subject exposure dependency. If a substantial discrepancy is found (e.g., more than a pre-specified threshold such as 5 to 10% of the Mantel-Haenszel odds ratio), standard conditional regression should not be used. Either the Mantel-Haenszel method or our weighting method should be used instead. The weighting method has less bias than the Mantel-Haenszel method when a time-varying confounder exists and in such a case we recommend using our proposed method to estimate *OR*_*G*_ in the analysis of case-crossover data because it removes bias from within-subject exposure dependency and can account for time-varying confounders.

Future research will extend our weighting method to allow for time trends in the exposure, ‘washout periods’, multi-exposure levels which are potentially important when the event rate changes during exposed periods, and analytical method to have the variance of *OR*_*G*_ by making allowance for the uncertainty in estimating the weight.

## Supplementary Information

**Additional file 1.**

## Data Availability

The datasets used and/or analyzed during the current study are available from the corresponding author on reasonable request.
