# Supplementary material for: A novel weighting method to remove bias from within-subject exposure dependency in case-crossover studies
Source: BMC Med Res Methodol. 2021 Oct 17;21:214. doi: 10.1186/s12874-021-01408-5 (PMC8520620; doi:10.1186/s12874-021-01408-5)
Supplement: Supplementary file 1 — Additional file 1. [file 12874_2021_1408_MOESM1_ESM.docx]

**Additional File 1**

A novel weighting method to remove bias from within-subject exposure dependency in case-crossover studies

Kiyoshi Kubota, Lan Kelly, Tsugumichi Sato, Nicole Pratt, Elizabeth Roughead, Takuhiro Yamaguchi

**Note:** Appendix 4-4 shows SAS codes for a cyclic pattern of 2 exposed periods and 1 unexposed period given in DRUG TREATMENT WITH SPECIFIC EXPOSURE PATTERN of Background section of the text.

**Contents**

**Appendix 1** Standard conditional logistic regression for the case-crossover study when the exposure is stationary and occurs independently between observed periods **P2**

**Appendix 2** Maximum likelihood estimate of exp(β) in Equation (12) **P4**

**Appendix 3** The likelihood for a binary exposure variable with within-subject dependency and a binary time-varying confounder without within-subject dependency **P7**

**Appendix 4** SAS codes for creating and analyzing simulated data and results **P9**

**Appendix 4-1** SAS codes for creating and analyzing simulated data with a binary exposure variable only (Figure 2a) to produce results in Table 2 (1 period=1 day) **p13**

**Appendix 4-2** SAS codes for creating and analyzing simulated data with a binary exposure variable and a binary confounder (Figure 2b) to produce results in Table 3 (1 period=1 day) **p22**

**Appendix 4-3** SAS codes for analyses of simulated data in Figure 2a and Figure 2b to produce results in Table 4 (1 period=2, 3, 4, 7, 11 days)  **P32**

**Appendix 4-4** SAS codes for creating and analyzing simulated data in the example in Background section in the text **P38**

**Appendix 5** Case-crossover study on the association between celecoxib and peripheral edema: a study using Japanese claims database **P39**

**Appendix 1**

**Standard conditional logistic regression for the case-crossover study when the exposure is stationary and occurs independently between observed periods**

In Appendix 1, we assume that a binary exposure is stationary (the exposure probability does not change) over the observed periods and occurs independently between periods i.e., within-subject exposure dependency does not occur.

When a binary exposure occurs independently between observed periods and the exposure probability is stationary, the exposure distribution follows a binomial distribution. For a study with 1 case period and M control periods, the probability that m of (M+1) periods is exposed, $P_{m}$, is given by:

$P_{m}=C(M+1,m)\pi^{m}{(1-\pi)}^{M-m+1}$ (A1)

where $\pi$ is the exposure probability at each period and $C(M+1,m)$ is a binomial coefficient. In the case-crossover study, concordant cases with m=0 or m=M+1 do not contribute to the estimation of the odds ratio (OR) and hereafter, m is considered to be either 1, 2, ---, or M. $P_{m}$ can be expressed as the sum of $P_{1,m}$ and $P_{0,m} \left( P_{m}=P_{1,m}+ P_{0,m} \right)$ where $P_{1,m}$ is the probability that the case-period is exposed and $P_{0,m}$ is the probability that the case-period is unexposed$,$such that:

$P_{1,m}=\pi C(M,m-1)\pi^{m-1}{(1-\pi)}^{M-m+1}$

and

$P_{0,m}=\left( 1-\pi\right)C\left( M,m \right)\pi^{m}\left( 1-\pi\right)^{M-m}$ (A2)

Since the focus of the current discussion is bias rather than random error, in order to assess whether the estimate is biased, we may assume that the number of exposed cases with m exposed periods, $a_{1,m}$, and the number of unexposed cases with m exposed periods, $a_{0,m}$, are equal to the expected number, or:

$a_{1,m}=Nr_{1}P_{1,m}$

and

$a_{0,m}=Nr_{0}P_{0,m}$ (A3)

where N is the population size and $r_{1}$ and $r_{0}$ are the rate of the outcome occurrence per period during the exposed and unexposed period, respectively. When the rate ratio, $RR$, is defined as $RR=r_{1}/r_{0}$, $a_{0,m}$ can be expressed as:

$a_{0,m}=a_{1,m}\frac{M-m+1}{m RR}$  (A4)

The likelihood for standard conditional logistic regression *L* in Equation (2) in the text can be expressed as $L=L_{1}L_{0}$ where $L_{1}$ is for the exposed cases and $L_{0}$ is for the unexposed cases given by:

$L_{1}=\prod_{m=1}^{M} \left( \frac{exp(\beta)}{m exp\left( \beta\right)+M-m+1} \right)^{a_{1,m}}$

and

$L_{0}=\prod_{m=1}^{M} \left( \frac{1}{m exp\left( \beta\right)+M-m+1} \right)^{a_{0,m}}$ (A5)

Let the logarithm of $L$, $L_{1}$ and $L_{0}$ be expressed as $l$, $l_{1}$ and $l_{0}$, respectively:

$l=\sum_{m=1}^{M} l_{m}$, $l_{1}=\sum_{m=1}^{M} l_{1m}$, and $l_{0}=\sum_{m=1}^{M} l_{0m}$ ($l_{m}=l_{1m}+l_{0m}$)

where

$l_{1m}=a_{1,m}\beta-a_{1,m}log(m exp\left( \beta\right)+M-m+1)$

and

$l_{0m}=-a_{0,m}log(m exp\left( \beta\right)+M-m+1)$ (A6)

Using these relationships in Equation (A6), the following is derived:

$\frac{dl_{m}}{d\beta}=\frac{dl_{1m}}{d\beta}+\frac{dl_{0m}}{d\beta}=a_{1,m}-\frac{\left( a_{1,m}+a_{0,m} \right)m exp(\beta)}{m exp\left( \beta\right)+M-m+1}$ (A7)

To solve $\frac{dl_{m}}{d\beta}$=0, we have

$\exp\left( \beta\right)=\frac{a_{1,m}}{a_{0,m}}\left( M-m+1 \right)/m=RR$ (A8)

This relationship holds for all values of m (1, 2, ---, M). Therefore,$\exp\left( \beta\right)$ that satisfies the relationship $\frac{dl}{d\beta}=\sum_{m} \frac{dl_{m}}{d\beta}=0$ is given by $\exp\left( \beta\right)$ = $RR$. Hence, when there are no within-subject exposure dependency and no exposure time trends, standard conditional logistic regression provides unbiased estimates of the rate ratio.

**Appendix 2**

**Maximum likelihood estimate of exp(β) in Equation (12)**

The coefficient $\beta$ that maximizes L in Equation (12) in the text maximizes L1 below.

$L1=\frac{1}{\left( 1+exp(\beta)\pi_{10} \right)^{a_{0}}}\frac{\left( exp(\beta) \right)^{a_{1}}}{\left( 1+exp(\beta)\pi_{10} \right)^{A-a_{0}}}$ (B1)

where A =$a_{0}$+$a_{1}$. The logarithm of L1 ($l$) is given by:

$l=a_{1}\beta-A log(1+exp(\beta)\pi_{10})$ (B2)

Thus when $\pi_{10}$ is considered to be a constant,

$\frac{dl}{d\beta}=a_{1}-\frac{A \pi_{10}exp(\beta)}{1+exp(\beta)\pi_{10}}$ (B3)

By solving $dl/d\beta=0$, we obtain Equation (13) ($\exp\left( \beta\right)=\frac{a_{1}}{a_{0}}\frac{1}{\pi_{10}}$).

By further differentiating $dl/d\beta$ in Equation (B3), we obtain:

$\frac{d^{2}l}{{d\beta}^{2}}=-\frac{A\pi_{10}exp(\beta)}{1+exp(\beta)\pi_{10}}+\frac{A{(\pi_{10}exp(\beta))}^{2}}{\left( 1+exp(\beta)\pi_{10} \right)^{2}}$

$=\frac{A\pi_{10}exp(\beta)}{\left( 1+exp(\beta)\pi_{10} \right)^{2}} \left( -1-\pi_{10}exp(\beta)+\pi_{10}exp(\beta) \right)$

$=-\frac{A\pi_{10}exp(\beta)}{\left( 1+exp(\beta)\pi_{10} \right)^{2}}$ (B4)

Using $E(\exp\left( \beta\right))=\frac{a_{1}}{a_{0}}\frac{1}{\pi_{10}}$, the information matrix I for 1 element ($=-E\left[ \frac{d^{2}l}{{d\beta}^{2}} \right]$) is given by:

$I=\frac{a_{1}a_{0}}{A}$ (B5)

The variance of $\beta$ is the reciprocal of I in Equation (B5) which is Equation (14) in the text as follows:

$v\left( \beta\right)=I^{-1}=\frac{A}{a_{1}a_{0}}=\frac{a_{1+}a_{0}}{a_{1}a_{0}}=\frac{1}{a_{0}}+\frac{1}{a_{1}}$ (B6)

It may be noted that the variance in Equation (B6) and Equation (14) in the text was derived by assuming that $\pi_{10}$ is a constant. We recommend estimating 2.5 to 97.5 percentiles of $exp(\beta)$ by bootstrap method rather than estimating 95% CI from a single dataset.

**Appendix 3**

**The likelihood for a binary exposure variable with within-subject dependency and a binary time-varying confounder without within-subject dependency**

In Appendix 3, we consider a case-crossover study with a binary exposure (x) and a binary time-varying confounder (z). We assume that both of them are stationary over the study period (i.e., no time trends). We also assume that the exposure variable may have within-subject dependency, but the time-varying confounder has no within-subject dependency i.e., the time-varying confounder in one period is independent of the exposure or confounder at different periods.

In this appendix, we provide the reason why the estimates of $\exp\left( \beta\right)$ and $\exp\left( \gamma\right)$ are unbiased when the likelihood in Equation (11) in the text is used in such a circumstance. To do this, in Appendix 3-1 below, we first explain why the estimate of $\exp\left( \beta\right)$ is unbiased when the likelihood in Equation (2) for the standard conditional regression is used when the exposure has no time trend and no within-subject dependency in a way which is different from Appendix 1. We next discuss the relationship between the likelihood in Equation (1) and that in Equation (3) in Appendix 3-2. Finally, we explain why the estimates of $\exp\left( \beta\right)$ and $\exp\left( \gamma\right)$ are unbiased by Equation (11) in Appendix 3-3.

**Appendix 3-1**

**Alternative explanation why Equation (2) yields unbiased estimates when a binary exposure is stationary and occurs independently between observation periods**

As explained in the text, for a case-crossover study with a binary exposure variable, the use of the likelihood in Equation (1) by Vines and Farrington can provide an unbiased estimate with or without within-subject dependency (and with or without time trends in the exposure). As in the text, when $X_{im}$ in Equation (1) denotes a binary exposure, the denominator of the likelihood in Equation (1) becomes $exp(\beta)\sum_{\kappa_{1}} P\left( X_{i0}=1, N_{exposed}=k \right) +\sum_{\kappa_{0}} P(X_{i0}=0, N_{exposed}=k)$.

When there is no time trend of the exposure and the exposure variable is independent between study periods, the probabilities of all (M+1)! permutations are the same (i.e., global exchangeability holds) and equal to 1/(M+1)!. In this case, $\sum_{\kappa_{1}} P\left( X_{i0}=1, N_{exposed}=k \right)=k/(M+1)$ and $\sum_{\kappa_{0}} P(X_{i0}=0, N_{exposed}=k)=(M+1-k)/(M+1)$ because the number of permutations where one of k exposed periods is located at the case period is $k M!$ and that where one of (M+1-k) unexposed periods is located at the case period is $(M+1-k)M!$. Thus, the denominator of Equation (1) becomes $\left( \exp\left( \beta\right)k+\left( M+1-k \right) \right)/(M+1)$ and as shown by Vines and Farrington when global exchangeability holds, the likelihood for a case-crossover study in Equation (1) is equivalent to that in Equation (2) and the use of Equation (2) provides the unbiased estimator of $exp(\beta)$.

**Appendix 3-2**

**Relationship between the Vines and Farrington's likelihood in Equation (1) and that in Greenland's likelihood in Equation (3)**

When within-subject exposure dependency is not satisfied, the estimate of $\exp\left( \beta\right)$ may be biased when the likelihood for the standard conditional logistic regression in Equation (2) is used, but if the probability that the individual is exposed ($\pi_{1}$) and that unexposed ($\pi_{0}$) at the case period and their ratio $\pi_{10}$ (${=\pi}_{1}/\pi_{0}$) in the population can be estimated (e.g., as in Equation (9) in the text), an unbiased estimate of $\exp\left( \beta\right)$ can be estimated when $\sum_{\kappa_{1}} P\left( X_{i0}=1, N_{exposed}=k \right)$ in the denominator of the likelihood in Equation (1) for a binary exposure variable is substituted by $\pi_{1}$ and $\sum_{\kappa_{0}} P(X_{i0}=0, N_{exposed}=k)$ is substituted by $\pi_{0}$ (or, the former is substituted by $\pi_{10}$ and the latter is substituted by 1) which is equivalent to the likelihood in Equation (3). This is also equivalent to the use of the likelihood for the standard conditional logistic regression with the weight in Equation (10) as shown in Equation (11) in the text.

**Appendix 3-3**

**Explanation why the use of the likelihood in Equation (11) yields the unbiased estimates when a binary exposure and binary time-varying confounder are stationary, and the latter occurs independently during exposed and unexposed periods**

In Appendix 3-3 to discuss a case-crossover study with a binary exposure (x) and binary time-varying confounder (z), we rewrite the number of exposed periods (previously denoted as m) as $m^{1}$ and the number of unexposed periods (M+1-m) as $m^{0}$ ($m^{1}+m^{0}=M+1$). For each individual i, in a case-crossover study, (M+1) periods may be divided into $m_{i}^{1}$ exposed and $m_{i}^{0}$ unexposed periods, $m_{i}^{1}$ exposed periods are further divided into $m_{i}^{11}$ confounder-positive periods and $m_{i}^{10}$ confounder-negative periods, and $m_{i}^{0}$ unexposed periods into $m_{i}^{01}$ confounder-positive periods and $m_{i}^{00}$ confounder-negative periods. For each individual i, $\beta x_{ij}$ in Equation (1) can be expressed as $(\beta, \gamma){(x_{ij}, z_{ij})}^{T}$ where $x_{ij}$ (j=0, 1, ---, M) is the exposure status at the j-th period and $z_{ij}$ (j=0, 1, ---, M) is the status of time-varying confounder at the j-th period. Then the denominator in Equation (1) in the text may be rewritten as the sum of $Q_{i11}$, $Q_{i10}$, $Q_{i01}$, and $Q_{i00}$ described below. Similarly, the numerator in Equation (1) is either of these 4 quantities corresponding to the status of x and z at the case period (i.e., $x_{i0}$ and $z_{i0}$).

$Q_{i11}=exp(\beta)exp(\gamma)\sum_{\kappa_{1}} P\left( x_{i0}=1, m_{i}^{1} \right) \sum_{\kappa_{11}} P\left( z_{i0}=1, m_{i}^{11}|m_{i}^{1} \right)$

$Q_{i10}=\exp\left( \beta\right)\sum_{\kappa_{1}} P\left( x_{i0}=1, m_{i}^{1} \right) \sum_{\kappa_{10}} P\left( z_{i0}=0, m_{i}^{11}|m_{i}^{1} \right)$

$Q_{i01}=\exp\left( \gamma\right)\sum_{\kappa_{0}} P\left( x_{i0}=0, m_{i}^{1} \right) \sum_{\kappa_{01}} P\left( z_{i0}=1, m_{i}^{01}|m_{i}^{0} \right)$

$Q_{i00}=\sum_{\kappa_{0}} P\left( x_{i0}=0, m_{i}^{1} \right) \sum_{\kappa_{00}} P\left( z_{i0}=0, m_{i}^{01}|m_{i}^{0} \right)$ (C1)

where$exp(\beta)$ is the rate ratio for the exposure variable ($RR$), $exp(\gamma)$ is the rate ratio for the time-varying confounder (${RR}_{z}$), $\sum_{\kappa_{1}} P\left( x_{i0}=1, m_{i}^{1} \right)$and $\sum_{\kappa_{1}} P\left( x_{i0}=0, m_{i}^{1} \right)$are the sum of probabilities for all the permutations for $m_{i}^{1}$ exposed and$m_{i}^{0}$ unexposed periods where $x_{i0}=1$ (the case period is exposed) and that where $x_{i0}=0$ (the case period is unexposed), respectively, $\sum_{\kappa_{11}} P\left( z_{i0}=1, m_{i}^{11}|m_{i}^{1} \right)$and $\sum_{\kappa_{10}} P\left( z_{i0}=0, m_{i}^{11}|m_{i}^{1} \right)$are the sum of probabilities for all permutations for $m_{i}^{11}$ confounder-positive and $m_{i}^{10}$ confounder-negative periods during $m_{i}^{1}$ exposed periods where $z_{i0}=1$ and $z_{i0}=0$, respectively, and $\sum_{\kappa_{01}} P\left( z_{i0}=1, m_{i}^{01}|m_{i}^{0} \right)$and $\sum_{\kappa_{00}} P\left( z_{i0}=0, m_{i}^{01}|m_{i}^{0} \right)$are the sum of probabilities for all permutations for $m_{i}^{01}$ confounder-positive and $m_{i}^{00}$ confounder-negative periods during $m_{i}^{0}$ unexposed periods where $z_{i0}=1$ and $z_{i0}=0$, respectively.

It may be noted that when a time-varying confounder exists, the expected number of exposed cases ($a_{1}$) and that of unexposed cases ($a_{0}$) are given by:

$a_{1}=N\pi_{1} r_{00}RR\left( {f_{1}RR}_{z}+\left( 1-f_{1} \right) \right)$

$a_{0}=N\pi_{0}r_{00} \left( {f_{0}RR}_{z}+\left( 1-f_{0} \right) \right)$ (C2)

where N is the population size, $r_{00}$ is the incidence rate per period in those that are unexposed and confounder-negative at the case period, $f_{1}$ is the proportion of confounder-positive in the exposed and $f_{0}$ is the proportion of confounder-positive in the unexposed, $RR$ is the rate ratio for the exposure (x), and ${RR}_{z}$ is the rate ratio for the confounder (z). When there is no time-varying confounding, ${RR}_{z}$ can be set to 1 and Equation (C2) simplifies to :

$a_{1}=N\pi_{1} r_{00}RR$

$a_{0}=N\pi_{0}r_{00}$ (C3)

When x and z have no time trends and z in one period is independent of x and z at different periods, $\pi_{1}$, $\pi_{0}$, and $\pi_{10}$ are estimated without bias by Equation (9) using $a_{1}$ exposed cases and $a_{0}$ unexposed cases from Equation (C2). This is because pairwise exchangeability of the exposure variable is the condition for $\pi_{10}$ in Equation (9) in the text to be unbiased, and pairwise exchangeability of the exposure variable does not change with or without a time-varying confounder which is independent of the exposure. As suggested in Appendix 4-2, in such a situation $\sum_{\kappa_{1}} P\left( x_{i0}=1, m_{i}^{1} \right)$and $\sum_{\kappa_{0}} P\left( x_{i0}=0, m_{i}^{1} \right)$ may be substituted by $\pi_{1}$ and $\pi_{0}$, respectively to yield unbiased estimates. Then, the denominator in Equation (1) for individual i $Q_{i}$ (=$Q_{i11}+Q_{i10}+Q_{i01}+Q_{i00}$) may be described as:

$Q_{i}=\exp\left( \beta\right)\pi_{1}\left( \exp\left( \gamma\right)\sum_{\kappa_{11}} P\left( z_{i0}=1, m_{i}^{11}|m_{i}^{1} \right)+\sum_{\kappa_{10}} P\left( z_{i0}=0, m_{i}^{11}|m_{i}^{1} \right) \right)$

$+\pi_{0}\left( \exp\left( \gamma\right)\sum_{\kappa_{01}} P\left( z_{i0}=1, m_{i}^{01}|m_{i}^{0} \right)+\sum_{\kappa_{00}} P\left( z_{i0}=0, m_{i}^{01}|m_{i}^{0} \right) \right)$ (C4)

Because the time-varying confounder is independent during $m_{i}^{1}$ exposed and $m_{i}^{0}$ unexposed periods, respectively, then according to the finding in Appendix 4-1, $\sum_{\kappa_{11}} P\left( z_{i0}=1,m_{i}^{11}|m_{i}^{1} \right)$=$m_{i}^{11}{/m}_{i}^{1}$, $\sum_{\kappa_{10}} P\left( z_{i0}=0, m_{i}^{11}|m_{i}^{1} \right)$=$m_{i}^{10}{/m}_{i}^{1}$, $\sum_{\kappa_{01}} P\left( z_{i0}=1, m_{i}^{01}|m_{i}^{0} \right)=m_{i}^{01}/m_{i}^{0}$, and $\sum_{\kappa_{00}} P\left( z_{i0}=0, m_{i}^{01}|m_{i}^{0} \right)$=$m_{i}^{00}/m_{i}^{0}$.

Substituting into Equation (D4), $Q_{i}$ (divided by $\pi_{0}$) can be rewritten as:

$Q_{i}/\pi_{0}=(\exp\left( \beta\right)\exp\left( \gamma\right)m_{i}^{11}+$ $\exp\left( \beta\right)m_{i}^{10})\pi_{10}/ m_{i}^{1}$ + $(exp \left( \gamma\right)m_{i}^{01}+$ $m_{i}^{00}$)$/m_{i}^{0}$ (C5)

From Equation (10) in the text, $w_{ij}$ = $\pi_{10}/m_{i}^{1}$ for exposed periods and $1/m_{i}^{0}$ for unexposed periods. Therefore Equation (C5) is equivalent to the denominator of the likelihood in Equation (11) ) for a binary exposure variable and binary time-varying confounder $\beta x_{ij}$=$(\beta, \gamma){(x_{ij}, z_{ij})}^{T}$.

Analysis of a case-crossover study with a binary exposure and time-varying confounder using conditional logistic regression involves calculating $\pi_{10}$ (${=\pi}_{1}/\pi_{0}$) using the exposure variable (x) only by Equation (9). Then data is analyzed by standard conditional logistic regression with weights from Equation (10) (See SAS codes 4-2e and 4-2f in Appendix 4).

If both x and z have within-subject dependency, the combination of (x, z) is regarded as a new "exposure" variable with 4 levels and the weights should be calculated for 4 levels. Methods to deal with exposures with multiple levels will be the subject of future publications.

**Appendix 4**

**SAS codes for creating and analyzing simulated data and results**

In Appendix 4, SAS codes for creating and analyzing simulated data, and results are shown. In Appendices 4-1 to 4-4, simulation is for a cyclic treatment pattern with stoppers and starters as in Figures 1 and 2 in the text. In Appendix 4-5, simulation is for a cyclic treatment pattern without stoppers and starters as mentioned in Background section in the text.

In Appendices 4-1 to 4-4, the exposure pattern depicts a dynamic population where the majority of patients are being treated but some patients stop, while some patients newly start the treatment. Under such an exposure pattern, standard conditional logistic regression gives a biased odds ratio, and the bias increases when the number of control periods (M) increases. We also demonstrate that even when the time-varying confounding exists, the unbiased odds ratio can be estimated by the weighting method proposed in the current study while the Mantel-Haenszel odds ratio ignoring the time-varying confounder is biased.

In Appendix Figure 4a below, a hypothetical population of patients for Appendices 4-1 to 4-4 is illustrated where they receive drug treatment with a cyclic pattern of 7 days. Patients take a drug at days 1 and 4 but not at days 2, 3, 5, 6, and 7. In the data given in Appendices 4-1, 4-2 and 4-4, 1 period is 1 day while in Appendices 4-3, 1 period is 2, 3, 4, 7, or 11 days. In Appendices 4-1 to 4-4, c0 indicates the exposure status at the case period and cm indicates that at the m-th control period at t (t=-m: m=1, 2, ---, 7). The patients can be divided into 8 subgroups of N=10,000 patients each. Of these 8 subgroups, patients in Subgroups B to G are being treated, while those in Subgroup A stopped the treatment at the case period and those in Subgroup H started the treatment at the case period. In Appendix Figure 4a, within-subject exposure dependency exists because exposure in one period is dependent on exposure in the preceding period(s) (e.g., an exposed period always follows an unexposed period). However, there is no time trend in the exposure because the proportion of the exposed is 1 in 4 for all of the 8 observation periods. It may be noted that the proportion of the exposed is 1 in 4 and the stationarity is maintained even one or more days pass from the day depicted by Appendix Figure 4a.

**Appendix Figure 4a** A hypothetical exposure pattern in patients receiving a cyclic drug treatment. Subgroup A has just stopped, Subgroup H has just started the treatment, and Subgroups B to G are currently being treated at the case period. Each subgroup consists of N=10,000 patients.

c0: exposure status at the case period; cm (m=1, 2, ---, 7): exposure status at the m-th control period.

In Appendix Figure 4a above, patients have 1 case period and 7 control periods, but using the data of 7 control periods in Appendix Figure 4a, the pattern of 15 periods with the same cyclic pattern can be generated by adding 1 set of control periods where c1, c2, --, c7 are renamed as c8, c9, --, c14, respectively to the pattern of 8 periods. Similarly, the pattern of 22 periods with the same cyclic pattern can be generated by further adding 1 set of control periods where c1, c2, --, c7 in Appendix Figure 4a are renamed as c15, c16, --, c21, respectively to the pattern of 15 periods. Figure 1 in the text is the pattern with 22 periods created in this way.

In the population in Appendix Figure 4a (and Figure 1 in the text), on average, 140 cases have an outcome at the case period (as shown in Appendix Figure 4b below and Figure 2a in the text) if the rate of outcome occurrence is 0.001 and 0.004 per period when the period is unexposed and exposed, respectively, and the rate ratio (RR) is 4.0.

Similarly, in the population in Appendix Figure 4a (and Figure 1 in the text), on average 184 cases have an outcome at the case period (as shown in Appendix Figure 4c below and Figure 2b in the text) if the rate of outcome occurrence is 0.001 when the period is unexposed and the status of time-varying confounder is negative, the rate ratio for the exposure (RR) is 4.0, that for the confounder (RRz) is 2.0 (without multiplicative interaction), and the proportion of the time-varying confounder in the unexposed (f0) is 0.2 and that in the exposed (f1) is 0.4. 　 　 Though the number of cases was assumed to be as expected, the confounder status in control periods (c1 to c21) was randomly generated as in SAS codes 4-2b to 4-2f.

| **ID_case** | **ID_subgroup** | **c0** | **n** |
| --- | --- | --- | --- |
| **1-10** | A | 0 | 10 |
| **11-20** | B | 0 | 10 |
| **21-30** | C | 0 | 10 |
| **31-40** | D | 0 | 10 |
| **41-80** | E | 1 | 40 |
| **81-90** | F | 0 | 10 |
| **91-100** | G | 0 | 10 |
| **101-140** | H | 1 | 40 |

**Appendix Figure 4b** 140 cases who had an outcome at the case period in a hypothetical population in Appendix Figure 4a (and Figure 1 in the text). The number of cases is the same as that in Figure 2a in the text.

c0: exposure status at the case period.

**Appendix Figure 4c** 184 cases who had an outcome at the case period in a hypothetical population in Appendix Figure 4a (and Figure 1 in the text). The number of cases is the same as that in Figure 2b in the text.

c0: exposure status at the case period; z0: status of a time-varying confounder at the case period.

**SAS codes in Appendix 4**

SAS codes 4-1 are to create and analyze simulated data with a binary exposure variable only (Appendix Figure 4b and Figure 2a in the text). SAS codes 4-1a to 4-1c create data which is then analyzed by the standard conditional logistic regression (4-1d), the Vines and Farrington method (4-1e), the Mantel-Haenszel method (4-1f), the Greenland's method (4-1g), and bootstrap method to estimate 2.5 to 97.5 percentiles of OR by Greenland's method (4-1h).

SAS code 4-2 are to create and analyze simulated data with a binary exposure variable and a binary confounding (Appendix Figure 4c and Figure 2b in the text). SAS codes 4-2a create data which is then analyzed by the standard conditional logistic regression (4-2b), the Vines and Farrington method (4-2c), the Mantel-Haenszel method (4-2d), the Greenland's method (4-2e), and bootstrap method to estimate 2.5 to 97.5 percentiles of OR by Greenland's method (4-2f).

SAS codes 4-3 are to analyze data created by SAS codes 4-1 and 4-2 while 1 period =2, 3, 4, 7, and 11 days. The exposure status is defined as the status at the last day of the period as in Definition I in Table 1 in the text. In SAS-codes 4-3a, 1 period= 2days, and the exposure status at the case period (c0) is the same as that in Figure 1 while c2, c4, ---, c20 are selected and renamed as c1, c2, ---, c10, respectively. Using the exposure status of the population where 1 period=2 days made by SAS code 4-3a, 140 cases in Figure 2a (Appendix Figure 4b) are created by SAS codes 4-1c, and 184 cases in Figure 2b (Appendix Figure 4c) are created by SAS codes 4-2a, and analyzed by the standard conditional logistic regression (SAS codes 4-1d and 4-2b, respectively), the Vines and Farrington method (4-1e and 4-2c, respectively), the Mantel-Haenszel method (4-1f and 4-2d, respectively), the Greenland's method (4-1g and 4-2e, respectively), and bootstrap method to estimate 2.5 to 97.5 percentiles of OR by Greenland's method (4-1h and 4-2f, respectively).

SAS codes 4-3b shows the codes for data simulation and analysis where 1 period=3 days and c3, c6, ---, c18 in Figure 1 in the text are renamed as c1, c2, ---, c6, respectively and then analyzed as in SAS codes 4-3a. SAS codes 4-3c shows the codes for data simulation and analysis where 1 period=4 days and c4, c8, ---, c16 in Figure 1 in the text are renamed as c1, c2, --, c4, respectively and then analyzed. SAS codes 4-3d shows the codes for data simulation and analysis where 1 period=7 days and the status at control periods c7, and c14 in Figure 1 in the text are renamed as c1 and c2, respectively and then analyzed. SAS codes 4-3e shows the codes for data simulation and analysis where 1 period=11 days and a control periods c11 in Figure 1 in the text is renamed as c1 and then analyzed.

**Appendix 4-1 SAS codes for creating and analyzing simulated data with a binary exposure variable only (Figure 2a) to produce results in Table 2 (1 period=1 day)**

[SAS codes 4-1a] Exposure patterns at the case period and 7 control periods for the population with a cyclic drug treatment

**data** dcase_p; length ID_subgroup $ **1**;

input ID_subgroup c0;

cards;

A 0

B 0

C 0

D 0

E 1

F 0

G 0

H 1

;

**data** dcontrol_p; length ID_subgroup $ **1**;

input ID_subgroup c1 c2 c3 c4 c5 c6 c7;

cards;

A 0 0 0 1 0 0 1

B 0 0 1 0 0 1 0

C 0 1 0 0 1 0 0

D 1 0 0 1 0 0 0

E 0 0 1 0 0 0 1

F 0 1 0 0 0 1 0

G 1 0 0 0 1 0 0

H 0 0 0 0 0 0 0

;

[SAS codes 4-1b] Exposure pattern in the population with a case period and n (3) cycles of control periods where one cycle has 7 periods

*Ncycle: number of treatment cycles;

**%macro** dpattern(Ncycle);

%do i=**1** %to &Ncycle;

%if &i=**1** %then %do;

data dcontrol_p1; set dcontrol_p;

%end;

%else %do;

data dcontrol_p&i(drop=c1-c7); set dcontrol_p;

%do j=**1** %to **7**;

%let jj=%eval(&j+(&i-1)*7);

c&jj=c&j;

%end;

%end;

%end;

data dpattern; merge dcase_p dcontrol_p1-dcontrol_p&Ncycle; by ID_subgroup;

**%mend** dpattern;

%***dpattern***(**3**);

[SAS codes 4-1c] Cases who have an outcome at the case period where the rate ratio (RR) is 4

*NN: size of each subgroup, r0: incidence rate when unexposed, RR: incidence rate ratio, data_pattern: data set of exposure pattern in the population,

data set 'dpattern' is produced by [SAS codes 4-1b];

**%macro** dcase(NN, r0, RR, data_pattern);

data dcase_info; set &data_pattern;

if c0=**1** then n=&NN*&r0*&RR; else n=&NN*&r0;

data dcase_info; set dcase_info;retain nl **0**;

if _n_=**1** then nf=**1**; else nf=nl+**1**; nl=nf+n-**1**;

*dcase: 1 record is 1 case;

data dcase(drop=n nf nl); set dcase_info;

do id=nf to nl; output;

end;

**%mend** dcase;

%***dcase***(**10000**,**0.001**,**4**, dpattern);

[SAS codes 4-1d] Standard Conditional Logistic Regression

*M: the largest number of control periods, data_case: data set of cases,

data set 'dcase' is produced by [SAS codes 4-1c];

**%macro** SCL(M, data_case);

%do i=**1** %to &M;

* dperiod: 1 record is 1 period;

data dperiods; set &data_case;

case=**1**; ex=IFN(c0=**1**,**1**,**0**);output;

%do j=**1** %to &i;

case=**0**; ex=IFN(c&j=**1**,**1**,**0**); output;

%end;

proc logistic data=dperiods descending; model case=ex ; strata iD;

ods output oddsratios=OR_SCL;

data OR_SCL(keep= OR_SCL OR_SCL_L OR_SCL_U M); set OR_SCL;

M=&i;

OR_SCL=OddsRatioEst; OR_SCL_L=LowerCL; OR_SCL_U=UpperCL;

%if &i=**1** %then %do; data CXO_results1; set OR_SCL; %end;

%else %do; data CXO_results1; set CXO_results1 OR_SCL; %end;

%end;

**%mend** SCL;

%***SCL***(**21**, dcase);

[SAS codes 4-1e] The Vines and Farrington’s method

*M: the largest number of control periods, data_pattern: data set of exposure pattern in the population, data_case: data set of cases,

data sets 'dpattern' and 'dcase' are produced by [SAS codes 4-1b] and [SAS codes 4-1c], respectively;

**%macro** VF(M, data_pattern, data_case);

%do i=**1** %to &M;

*weight for Vines and Farrington estimated from the population data

Nex is the number of exposed periods;

data dvf; set &data_pattern;

Nex=sum(of c0-c&i);

proc freq data=dvf noprint; tables Nex*c0 / out=dvf1;

data dvf1(drop=count percent); set dvf1; wt_VF=percent/**100**;

data dvf_w0 dvf_w1; set dvf1; if c0=**0** then output dvf_w0; else output dvf_w1;

data dvf_w0(rename=(wt_VF=wtVF0)); set dvf_w0;

data dvf_w1(rename=(wt_VF=wtVF1)); set dvf_w1;

data dvf_list(keep=Nex); set dvf1;

proc sort data=dvf_list nodupkey; by Nex;

data dvf_wt; merge dvf_list (in=ina) dvf_w0 dvf_w1 ; by Nex; if ina=**1**;

data dvf_wt(drop=c0); set dvf_wt;

*cases;

data dcaseVF; set &data_case;

Nex=sum(of c0-c&i);

proc sort data=dcaseVF; by Nex;

data dcaseVF; merge dcaseVF(in=ina) dvf_wt; by Nex; if ina=**1**;

* dperiod: 1 record is 1 period;

data dperiods; set dcaseVF;

case=**1**; ex=IFN(c0=**1**,**1**,**0**);output;

%do j=**1** %to &i;

case=**0**; ex=IFN(c&j=**1**,**1**,**0**); output;

%end;

data dperiods; set dperiods;

*weight per exposed/unexposed period;

if ex=**0** then wtVF=wtVF0/(&i+**1**-Nex);

else wtVF=wtVF1/(Nex);

lwVF=log(wtVF);

proc logistic data=dperiods descending; model case=ex / offset=lwVF; strata ID;

ods output oddsratios=OR_VF;

data OR_VF(keep=OR_VF OR_VF_L OR_VF_U M); set OR_VF;

OR_VF=OddsRatioEst; OR_VF_L=LowerCL; OR_VF_U=UpperCL;

M=&i;

%if &i=**1** %then %do; data CXO_results2; set OR_VF; %end;

%else %do; data CXO_results2; set CXO_results2 OR_VF; %end;

%end;

**%mend** VF;

%***VF***(**21**, dpattern, dcase);

[SAS codes 4-1f]The Mantel-Haenszel method

*M: the largest N of control periods, data_case: data set of cases,

data set 'dcase' is produced by [SAS codes 4-1c];

**%****macro** MH(M, data_case);

%do i=**1** %to &M;

data dperiods; set &data_case;

case=**1**; ex=IFN(c0=**1**,**1**,**0**); output;

%do j=**1** %to &i;

case=**0**; ex=IFN(c&j=**1**,**1**,**0**); output;

%end;

*Mantel-Haenszel method;

proc freq data=dperiods noprint; tables id*case*ex / cmh;

output mhor out=OR_MH;

data OR_MH(keep= M OR_MH OR_MH_L OR_MH_U); set OR_MH;

OR_MH=_MHOR_; OR_MH_L=L_MHOR; OR_MH_U=U_MHOR;

M=&i;

%if &i=**1** %then %do; data CXO_results3; set OR_MH; %end;

%else %do; data CXO_results3; set CXO_results3 OR_MH; %end;

%end;

**%mend** MH;

%***MH***(**21**, dcase);

[SAS codes 4-1g] The Greenland’s method

*M: the largest number of control periods, data_case: data set of cases,

data set 'dcase' is produced by [SAS codes 4-1c];

**%macro** GL(M, data_case);

%do i=**1** %to &M;

*find concordant exposure pattern;

data dcase_g; set &data_case; by id; retain discordant;

if first.id then discordant=**0**;

%do j=**1** %to &i;

discordant=discordant+ifn(c0=c&j,**0**,**1**);

%end;

*delete concordant pattern;

data dcase_g; set dcase_g; if discordant>**0**;

dummy=**1**;

data dcase_g; set dcase_g;

N1=sum(of c1-c&i);

PT10=IFN(c0=**1**, &i-N1,**0**);

PT01=IFN(c0=**0**, N1, **0**);

PT1CXO=N1+c0;

PT0CXO=&i-N1+(**1**-c0);

dummy=**1**;

*Estimate of pi10 and pi00;

data dpi; set dcase_g; retain a0 **0** a1 **0** PT10m **0** PT01m **0**;

PT01m=PT01m+PT01;

PT10m=PT10m+PT10;

a1=a1+c0;

a0=a0+(**1**-c0);

data dpi; set dpi end=final;

if final then output;

data dpi(keep=dummy pi00 pi10); set dpi;

dummy=**1**;

*pi00 is pi0/pi0 and pi10=pi1/pi0;

PT10m=PT10m/a1;

PT01m=PT01m/a0;

pi00=**1**; pi10=PT01m/PT10m;

data dcase_g; merge dcase_g(in=ina) dpi(in=inb); by dummy; if ina=**1** and inb=**1**;

data dperiods; set dcase_g;

w0=pi00/PT0CXO;

w1=pi10/PT1CXO;

* dperiods: 1 record is 1 period;

data dperiods; set dperiods;

case=**1**; ex=IFN(c0=**1**,**1**,**0**); wt=IFN(c0=**1**, w1, w0); output;

%do j=**1** %to &i;

case=**0**; ex=IFN(c&j=**1**,**1**,**0**); wt=IFN(c&j=**1**, w1, w0); output;

%end;

data dperiods(keep=id case ex lw); set dperiods;

lw=log(wt);

*OR_G Greenland;

proc logistic data=dperiods descending ; model case=ex / offset=lw; strata id;

ods output oddsratios=OR_G;

data OR_G(keep= OR_G OR_G_L OR_G_U M); set OR_G;

OR_G=OddsRatioEst; OR_G_L=LowerCL; OR_G_U=UpperCL;

M=&i;

%if &i=**1** %then %do; data CXO_results4; set OR_G; %end;

%else %do; data CXO_results4; set CXO_results4 OR_G; %end;

%end;

**%mend** GL;

%***GL***(**21**, dcase);

[SAS codes 4-1h] Bootstrap method to estimate 2.5 to 97.5 percentiles of OR_G

*M: the largest number of control periods, data_case: data set of cases,

data set 'dcase' is produced by [SAS codes 4-1c];

**%macro** GL_bs(M, data_case);

%do i=**1** %to &M;

*find concordant exposure pattern;

data dcase_g; set &data_case; by id; retain discordant;

if first.id then discordant=**0**;

%do j=**1** %to &i;

discordant=discordant+ifn(c0=c&j,**0**,**1**);

%end;

*delete concordant pattern;

data dcase_g; set dcase_g; if discordant>**0**;

dummy=**1**;

Proc surveyselect data=dcase_g out=dcase_g_boot

Seed=**4321**

Method=urs

Samprate=**1**

outhits

Rep=**1000**;

*each observation should be a different case even if the same patient is re-sampled twice or more;

data dcase_G_boot(drop=id); set dcase_G_boot;

data dcase_G_boot; set dcase_G_boot; by replicate; retain id;

if first.replicate then id=**0**;

id=id+**1**;

data dcase_g_boot; set dcase_g_boot;

N1=sum(of c1-c&i);

PT10=IFN(c0=**1**, &i-N1,**0**);

PT01=IFN(c0=**0**, N1, **0**);

PT1CXO=N1+c0;

PT0CXO=&i-N1+(**1**-c0);

*Estimate of pi10 and pi00;

data dpi_boot; set dcase_g_boot; by replicate; retain a0 a1 PT10m PT01m;

if first.replicate then do; a0=**0**; a1=**0**; PT10m=**0**; PT01m=**0**; end;

PT01m=PT01m+PT01;

PT10m=PT10m+PT10;

a1=a1+c0;

a0=a0+(**1**-c0);

if last.replicate then output;

data dpi_boot(keep=replicate pi00 pi10); set dpi_boot;

*pi00 is pi0/pi0 and pi10=pi1/pi0;

PT10m=PT10m/a1;

PT01m=PT01m/a0;

pi00=**1**; pi10=PT01m/PT10m;

data dcase_g_boot; merge dcase_g_boot(in=ina) dpi_boot(in=inb); by replicate; if ina=**1** and inb=**1**;

data dperiods_boot; set dcase_g_boot;

w0=pi00/PT0CXO;

w1=pi10/PT1CXO;

* dperiods: 1 record is 1 period;

data dperiods_boot; set dperiods_boot;

case=**1**; ex=IFN(c0=**1**,**1**,**0**); wt=IFN(c0=**1**, w1, w0); output;

%do j=**1** %to &i;

case=**0**; ex=IFN(c&j=**1**,**1**,**0**); wt=IFN(c&j=**1**, w1, w0); output;

%end;

data dperiods_boot(keep=replicate id case ex lw); set dperiods_boot;

lw=log(wt);

*OR_G Greenland;

proc logistic data=dperiods_boot descending ; by replicate; model case=ex / offset=lw; strata id;

ods output oddsratios=OR_G_boot;

data OR_G_boot(keep= OR_G_bs); set OR_G_boot; OR_G_bs=OddsRatioEst;

proc univariate data=OR_G_boot noprint; var OR_G_bs; output out=OR_G_bs_out n=n nobs=nobs pctlpts=**2.5**, **50**, **97.5** pctlpre=OR_G;

data OR_G_bs_out; set OR_G_bs_out;

M=&i;

%if &i=**1** %then %do; data CXO_results4_2; set OR_G_bs_out; %end;

%else %do; data CXO_results4_2; set CXO_results4_2 OR_G_bs_out; %end;

%end;

data CXO_results4_2; format M OR_G2_5 OR_G50 OR_G97_5 n nobs; set CXO_results4_2;

**%mend** GL_bs;

%***GL_bs***(**21**, dcase);

[SAS codes 4-1i] Summary for 4 methods for data with a binary exposure only without time trend

**data** CXO_results_A; merge CXO_results1-CXO_results4 CXO_results4_2; by M ; **run**;

**Appendix 4-2 SAS codes for creating and analyzing simulated data with a binary exposure variable and a binary confounder (Figure 2b) to produce results in Table 3 (1 period=1 day)**

[SAS codes 4-2a] Cases who have an outcome at the case period where the rate ratio (RR) is 4, the rate ratio for binary confounder (RRz) is 2, and the proportion of a binary confounder in the exposed (f1) is 0.4, and that in the unexposed (f0)=0.2

*macro dpattern is given in [SAS codes 4-1b],

data set 'dpattern' is produced by [SAS codes 4-1b];

%***dpattern***(**3**);

**data** dpattern20; set dpattern; z0=**0**;

**data** dpattern21; set dpattern; z0=**1**;

**data** dpattern2; set dpattern21 dpattern20;

**proc** **sort** data=dpattern2; by ID_subgroup z0;

*NN: size of each subgroup, r0: incidence rate when unexposed and with no confounder,

RR: incidence rate ratio for the exposure, RRz: incidence rate ratio for the confounder,

f0: proportion of confounder in the unexposed, f1: proportion of confounder in the exposed;

**%macro** dcase2(NN, r0, RR, RRz, f0, f1);

data dcase_info2; set dpattern2;

if c0=**1** and z0=**1** then n=&NN*&r0*&RR*&RRz*&f1;

else if c0=**1** and z0=**0** then n=&NN*&r0*&RR*(**1**-&f1);

else if c0=**0** and z0=**1** then n=&NN*&r0*&RRz*&f0;

else n=&NN*&r0*(**1**-&f0);

data dcase_info2; set dcase_info2; retain nl **0**;

if _n_=**1** then nf=**1**; else nf=nl+**1**; nl=nf+n-**1**;

data dcase2(drop=n nf nl); set dcase_info2;

do id=nf to nl; output;

end;

**%mend** dcase2;

%***dcase2***(**10000**,**0.001**, **4**, **2**, **0.2**, **0.4**);

[SAS codes 4-2b] Standard Conditional Logistic Regression with a time-varying binary confounder

*M: the largest number of control periods, data_case: data set of cases,

data set 'dcase2' is produced by [SAS codes 4-2a];

**%macro** SCL2(M, f0, f1, data_case);

%do i=**1** %to &M;

* dperiod: 1 record is 1 period;

data dperiods; set &data_case;

case=**1**; ex=IFN(c0=**1**,**1**,**0**);z=IFN(z0=**1**,**1**,**0**); output;

%do j=**1** %to &i;

%let k=%eval(43210+&j);

call streaminit(&k);

x=rand('uniform');

if c&j=**1** and x<=&f1 then do; case=**0**; ex=**1**; z=**1**; output; end;

else if c&j=**1** and x>&f1 then do; case=**0**; ex=**1**; z=**0**; output; end;

else if c&j=**0** and x<=&f0 then do; case=**0**; ex=**0**; z=**1**; output; end;

else do; case=**0**; ex=**0**; z=**0**; output; end;

%end;

*Standard conditional logistic regression;

proc logistic data=dperiods descending; model case=ex z ; strata iD;

ods output oddsratios=OR_SCL;

data OR_SCL(keep=variable OR_SCL OR_SCL_L OR_SCL_U M); set OR_SCL;

M=&i; variable=effect;

OR_SCL=OddsRatioEst; OR_SCL_L=LowerCL; OR_SCL_U=UpperCL;

%if &i=**1** %then %do; data CXO_results5; set OR_SCL; %end;

%else %do; data CXO_results5; set CXO_results5 OR_SCL; %end;

%end;

**%mend** SCL2;

%***SCL2***(**21**, **0.2**, **0.4**, dcase2);

[SAS codes 4-2c] The Vines and Farrington’s method with a time-varying binary confounder

*M: the largest number of control periods, *f0: proportion of confounder in the unexposed, f1: proportion of confounder in the exposed,

data_pattern: data set of exposure pattern in the population, data_case: data set of cases,

data sets 'dpattern' and 'dcase2' are produced by [SAS codes 4-1b] and [SAS codes 4-2a], respectively;

**%macro** VF2(M, f0, f1, data_pattern, data_case);

%do i=**1** %to &M;

*weight for Vines and Farrington estimated from the population data

Nex is the number of exposed periods;

data dvf; set &data_pattern;

Nex=sum(of c0-c&i);

proc freq data=dvf noprint; tables Nex*c0 / out=dvf1;

data dvf1(drop=count percent); set dvf1; wt_VF=percent/**100**;

data dvf_w0 dvf_w1; set dvf1; if c0=**0** then output dvf_w0; else output dvf_w1;

data dvf_w0(rename=(wt_VF=wtVF0)); set dvf_w0;

data dvf_w1(rename=(wt_VF=wtVF1)); set dvf_w1;

data dvf_list(keep=Nex); set dvf1;

proc sort data=dvf_list nodupkey; by Nex;

data dvf_wt; merge dvf_list (in=ina) dvf_w0 dvf_w1 ; by Nex; if ina=**1**;

data dvf_wt(drop=c0); set dvf_wt;

*cases;

data dcaseVF2; set &data_case;

Nex=sum(of c0-c&i);

proc sort data=dcaseVF2; by Nex;

data dcaseVF2; merge dcaseVF2(in=ina) dvf_wt; by Nex; if ina=**1**;

* dperiod: 1 record is 1 period;

data dperiods; set dcaseVF2;

case=**1**; ex=IFN(c0=**1**,**1**,**0**);z=IFN(z0=**1**,**1**,**0**); output;

%do j=**1** %to &i;

%let k=%eval(43210+&j);

call streaminit(&k);

x=rand('uniform');

if c&j=**1** and x<=&f1 then do; case=**0**; ex=**1**; z=**1**; output; end;

else if c&j=**1** and x>&f1 then do; case=**0**; ex=**1**; z=**0**; output; end;

else if c&j=**0** and x<=&f0 then do; case=**0**; ex=**0**; z=**1**; output; end;

else do; case=**0**; ex=**0**; z=**0**; output; end;

%end;

data dperiods; set dperiods;

*weight per exposed/unexposed period;

if ex=**0** then wtVF=wtVF0/(&i+**1**-Nex);

else wtVF=wtVF1/(Nex);

lwVF=log(wtVF);

proc logistic data=dperiods descending; model case=ex z/ offset=lwVF; strata ID;

ods output oddsratios=OR_VF;

data OR_VF(keep=variable OR_VF OR_VF_L OR_VF_U M); set OR_VF;

variable=effect;

OR_VF=OddsRatioEst; OR_VF_L=LowerCL; OR_VF_U=UpperCL;

M=&i;

%if &i=**1** %then %do; data CXO_results6; set OR_VF; %end;

%else %do; data CXO_results6; set CXO_results6 OR_VF; %end;

%end;

**%mend** VF2;

%***VF2***(**21**, **0.2**, **0.4**, dpattern, dcase2);

[SAS codes 4-2d] The Mantel-Haenszel method with a time-varying binary confounder

*M: the largest number of control periods, *f0: proportion of confounder in the unexposed, f1: proportion of confounder in the exposed,

data_case: data set of cases, data set 'dcase2' is produced by [SAS codes 4-2a];

**%macro** MH2(M, f0, f1, data_case);

%do i=**1** %to &M;

data dperiods; set &data_case;

* dperiods: 1 record is 1 period;

data dperiods; set dperiods;

case=**1**; ex=IFN(c0=**1**,**1**,**0**); z=IFN(z0=**1**,**1**,**0**); output;

%do j=**1** %to &i;

%let k=%eval(43210+&j);

call streaminit(&k);

x=rand('uniform');

if c&j=**1** and x<=&f1 then do; case=**0**; ex=**1**; z=**1**; output; end;

else if c&j=**1** and x>&f1 then do; case=**0**; ex=**1**; z=**0**; output; end;

else if c&j=**0** and x<=&f0 then do; case=**0**; ex=**0**; z=**1**; output; end;

else do; case=**0**; ex=**0**; z=**0**; output; end;

%end;

*select case period;

data dcase_p(keep=id c0 z0 case); set dperiods; if case=**1**;

*select case period where z=0;

data dcase_pz0; set dcase_p; if z0=**0**;

*select case period where z=1;

data dcase_pz1; set dcase_p; if z0=**1**;

*select periods where z=0 for subjects with case period where z=0;

data dperiods_z0; merge dperiods (in=ina) dcase_pz0 (in=inb); by id; if ina=**1** and inb=**1**;

data dperiods_z0; set dperiods_z0; if z=**0**;

*select periods where z=1 for subjects with case period where z=1;

data dperiods_z1; merge dperiods (in=ina) dcase_pz1 (in=inb); by id; if ina=**1** and inb=**1**;

data dperiods_z1; set dperiods_z1; if z=**1**;

*Mantel-Haenszel method;

*OR for exposure in a stratum where z=0;

proc freq data=dperiods_z0 noprint; tables id*case*ex / cmh;

output mhor out=OR_MH_z0;

data OR_MH_z0(keep=variable OR_MH_z0 OR_MH_L_z0 OR_MH_U_z0); set OR_MH_z0;

variable="ex";

OR_MH_z0=_MHOR_; OR_MH_L_z0=L_MHOR; OR_MH_U_z0=U_MHOR;

*OR for exposure in a stratum where z=1;

proc freq data=dperiods_z1 noprint; tables id*case*ex / cmh;

output mhor out=OR_MH_z1;

data OR_MH_z1(keep=variable OR_MH_z1 OR_MH_L_z1 OR_MH_U_z1); set OR_MH_z1;

variable="ex";

OR_MH_z1=_MHOR_; OR_MH_L_z1=L_MHOR; OR_MH_U_z1=U_MHOR;

*OR for exposure stratified by z;

data dperiods_z; set dperiods_z0 dperiods_z1;

proc freq data=dperiods_z noprint; tables id*case*ex / cmh;

output mhor out=OR_MH_z01;

data OR_MH_z01(keep=variable OR_MH_z OR_MH_L_z OR_MH_U_z); set OR_MH_z01;

variable="ex";

OR_MH_z=_MHOR_; OR_MH_L_z=L_MHOR; OR_MH_U_z=U_MHOR;

*Mantel-Haenszel method ignoring time-varying confounder;

proc freq data=dperiods noprint; tables id*case*ex / cmh;

output mhor out=OR_MH;

data OR_MH(keep=variable OR_MH OR_MH_L OR_MH_U M); set OR_MH;

variable="ex";

OR_MH=_MHOR_; OR_MH_L=L_MHOR; OR_MH_U=U_MHOR;

M=&i;

data OR_MH; merge OR_MH_z0 OR_MH_z1 OR_MH_z01 OR_MH;

%if &i=**1** %then %do; data CXO_results7; set OR_MH; %end;

%else %do; data CXO_results7; set CXO_results7 OR_MH; %end;

%end;

**%mend** MH2;

%***MH2***(**21**, **0.2**, **0.4**, dcase2);

[SAS codes 4-2e] The Greenland’s method with a time-varying binary confounder

*M: the largest number of control periods, *f0: proportion of confounder in the unexposed, f1: proportion of confounder in the exposed,

data_case: data set of cases, data set

'dcase2' is produced by [SAS codes 4-2a];

**%macro** GL2(M, f0, f1, data_case);

%do i=**1** %to &M;

*find concordant exposure pattern;

data dcase_g2; set &data_case; by id; retain discordant;

if first.id then discordant=**0**;

%do j=**1** %to &i;

discordant=discordant+ifn(c0=c&j,**0**,**1**);

%end;

* concordant patterns useful to estimate coefficient of time-varying confounder are not deleted when time-varying confounder exists;

data dcase_g2; set dcase_g2;

dummy=**1**;

data dcase_g2; set dcase_g2;

N1=sum(of c1-c&i);

PT10=IFN(c0=**1**, &i-N1,**0**);

PT01=IFN(c0=**0**, N1, **0**);

PT1CXO=N1+c0;

PT0CXO=&i-N1+(**1**-c0);

dummy=**1**;

*Estimate of pi10 and pi00;

data dpi; set dcase_g2; retain a0 **0** a1 **0** PT10m **0** PT01m **0**;

PT01m=PT01m+PT01;

PT10m=PT10m+PT10;

if discordant>**0** then do;

a1=a1+c0;

a0=a0+(**1**-c0);

end;

data dpi; set dpi end=final;

if final then output;

data dpi(keep=dummy pi00 pi10); set dpi;

dummy=**1**;

*pi00 is pi0/pi0 and pi10=pi1/pi0;

PT10m=PT10m/a1;

PT01m=PT01m/a0;

pi00=**1**; pi10=PT01m/PT10m;

data dcase_g2; merge dcase_g2(in=ina) dpi(in=inb); by dummy; if ina=**1** and inb=**1**;

data dperiods; set dcase_g2;

if PT0CXO=**0** then w0=**.**;

else w0=pi00/PT0CXO;

if PT1CXO=**0** then w1=**.**;

else w1=pi10/PT1CXO;

* dperiods: 1 record is 1 period;

data dperiods; set dperiods;

case=**1**; ex=IFN(c0=**1**,**1**,**0**); z=IFN(z0=**1**,**1**,**0**); wt=IFN(c0=**1**, w1, w0); output;

%do j=**1** %to &i;

%let k=%eval(43210+&j);

call streaminit(&k);

x=rand('uniform');

if c&j=**1** and x<=&f1 then do; case=**0**; ex=**1**; z=**1**; wt=w1; output; end;

else if c&j=**1** and x>&f1 then do; case=**0**; ex=**1**; z=**0**; wt=w1; output; end;

else if c&j=**0** and x<=&f0 then do; case=**0**; ex=**0**; z=**1**; wt=w0; output; end;

else do; case=**0**; ex=**0**; z=**0**; wt=w0;output; end;

%end;

data dperiods; set dperiods;

lw=log(wt);

*OR_G Greenland;

proc logistic data=dperiods descending ; model case=ex z/ offset=lw; strata id;

ods output oddsratios=OR_G;

data OR_G(keep=variable OR_G OR_G_L OR_G_U M); set OR_G;

variable=effect;

OR_G=OddsRatioEst; OR_G_L=LowerCL; OR_G_U=UpperCL;

M=&i;

%if &i=**1** %then %do; data CXO_results8; set OR_G; %end;

%else %do; data CXO_results8; set CXO_results8 OR_G; %end;

%end;

**%mend** GL2;

%***GL2***(**21**, **0.2**, **0.4**, dcase2);

[SAS codes 4-2f] Bootstrap method to estimate 2.5 to 97.5 percentiles of OR_G for data with a binary exposure and a binary confounder without time trend

*M: the largest number of control periods, *f0: proportion of confounder in the unexposed, f1: proportion of confounder in the exposed,

data_case: data set of cases, data set

'dcase2' is produced by [SAS codes 4-2a];

**%macro** GL_bs2(M, f0, f1, data_case);

%do i=**1** %to &M;

*find concordant exposure pattern;

data dcase_g2; set &data_case; by id; retain discordant;

if first.id then discordant=**0**;

%do j=**1** %to &i;

discordant=discordant+ifn(c0=c&j,**0**,**1**);

%end;

* concordant patterns useful to estimate coefficient of time-varying confounder are not deleted when time-varying confounder exists;

data dcase_g2; set dcase_g2;

dummy=**1**;

data dcase_g2; set dcase_g2;

N1=sum(of c1-c&i);

PT10=IFN(c0=**1**, &i-N1,**0**);

PT01=IFN(c0=**0**, N1, **0**);

PT1CXO=N1+c0;

PT0CXO=&i-N1+(**1**-c0);

data dcase_g2; set dcase_g2;

%do j=**1** %to &i;

%let k=%eval(43210+&j);

call streaminit(&k);

x=rand('uniform');

if c&j=**1** and x<=&f1 then z&j=**1**;

else if c&j=**1** and x>&f1 then z&j=**0**;

else if c&j=**0** and x<=&f0 then z&j=**1**;

else z&j=**0**;

%end;

*resmples for bootstrap;

Proc surveyselect data=dcase_g2 out=dcase_g_boots

Seed=**4321**

Method=urs

Samprate=**1**

outhits

Rep=**1000**;

*each observation should be a different case even if the same patient is re-sampled twice or more;

data dcase_G_boots (drop=id); set dcase_G_boots;

data dcase_G_boots; set dcase_G_boots; by replicate; retain id;

if first.replicate then id=**0**;

id=id+**1**;

*Estimate of pi10 and pi00;

data dpi_boot; set dcase_g_boots; by replicate; retain a0 a1 PT10m PT01m;

if first.replicate then do; a0=**0**; a1=**0**; PT10m=**0**; PT01m=**0**; end;

PT01m=PT01m+PT01;

PT10m=PT10m+PT10;

if discordant>**0** then do;

a1=a1+c0;

a0=a0+(**1**-c0);

end;

if last.replicate then output;

data dpi_boot(keep=replicate pi00 pi10); set dpi_boot;

*pi00 is pi0/pi0 and pi10=pi1/pi0;

PT10m=PT10m/a1;

PT01m=PT01m/a0;

pi00=**1**; pi10=PT01m/PT10m;

data dcase_g_boots; merge dcase_g_boots(in=ina) dpi_boot(in=inb); by replicate; if ina=**1** and inb=**1**;

data dperiods_boots; set dcase_g_boots;

if PT0CXO>**0** then w0=pi00/PT0CXO;

if PT1CXO>**0** then w1=pi10/PT1CXO;

* dperiods: 1 record is 1 period;

data dperiods_boots; set dperiods_boots;

case=**1**; ex=IFN(c0=**1**,**1**,**0**); z=IFN(z0=**1**, **1**, **0**); wt=IFN(c0=**1**, w1, w0); output;

%do j=**1** %to &i;

case=**0**; ex=IFN(c&j=**1**,**1**,**0**); z=IFN(z&j=**1**, **1**, **0**); wt=IFN(c&j=**1**, w1, w0); output;

%end;

data dperiods_boots(keep=replicate id case ex z lw); set dperiods_boots;

lw=log(wt);

*OR_G Greenland boot strap;

proc logistic data=dperiods_boots descending ; by replicate; model case=ex z/ offset=lw; strata id;

ods output oddsratios=OR_G_boots;

data OR_G_boots(keep=variable OR_G); set OR_G_boots; OR_G=OddsRatioEst;

variable=effect;

proc sort data=OR_G_boots; by variable;

proc univariate data=OR_G_boots noprint; by variable; var OR_G; output out=OR_G_bs_out n=n nobs=nobs pctlpts=**2.5**, **50**, **97.5** pctlpre=OR_G;

data OR_G_bs_out; set OR_G_bs_out;

M=&i;

%if &i=**1** %then %do; data CXO_results8_2; set OR_G_bs_out; %end;

%else %do; data CXO_results8_2; set CXO_results8_2 OR_G_bs_out; %end;

%end;

data CXO_results8_2; format M OR_G2_5 OR_G50 OR_G97_5 n nobs; set CXO_results8_2;

**%mend** GL_bs2;

%***GL_bs2***(**21**, **0.2**, **0.4**, dcase2);

[SAS codes 4-2g] Summary for 4 methods for data with a binary exposure and a binary confounder without time trend

**data** CXO_results_B; merge CXO_results5-CXO_results8 CXO_results8_2; by M variable; **run**;

**Appendix 4-3 SAS codes for analyses of simulated data in Figure 2a and Figure 2b to produce results in Table 4 (1 period=2, 3, 4, 7, 11 days)**

[SAS codes 4-3a] Analysis when 1 period=2days

*macros used are dcase [SAS codes 4-1c], SCL [4-1d], VF [4-1e], MH [4-1f] GL [4-1g] GL_bs[4-1h] ,dcase2 [4-2a], SCL2 [4-2b], VF2 [4-2c], MH2 [4-2d], GL2 [4-2e], and GL_bs2 [4-2f];

**data** dpattern3; length ID_subgroup $ **1**;

input ID_subgroup c0-c10;

cards;

A 0 0 1 0 0 0 0 1 0 1 0

B 0 0 0 1 0 1 0 0 0 0 1

C 0 1 0 0 0 0 1 0 1 0 0

D 0 0 1 0 1 0 0 0 0 1 0

E 1 0 0 0 0 1 0 1 0 0 0

F 0 1 0 1 0 0 0 0 1 0 1

G 0 0 0 0 1 0 1 0 0 0 0

H 1 0 0 0 0 0 0 0 0 0 0

;

%***dcase***(**10000**,**0.001**,**4**, dpattern3);

%***SCL***(**10**, dcase); %***VF***(**10**, dpattern3, dcase); %***MH***(**10**, dcase); %***GL***(**10**, dcase); %***GL_bs***(**10**, dcase);

**data** dpattern20; set dpattern3; z0=**0**;

**data** dpattern21; set dpattern3; z0=**1**;

**data** dpattern2; set dpattern21 dpattern20;

%***dcase2***(**10000**,**0.001**, **4**, **2**, **0.2**, **0.4**);

%***SCL2***(**10**, **0.2**, **0.4**, dcase2); %***VF2***(**10**, **0.2**, **0.4**, dpattern3, dcase2);

%***MH2***(**10**, **0.2**, **0.4**, dcase2); %***GL2***(**10**, **0.2**, **0.4**, dcase2); %***GL_bs2***(**10**, **0.2**, **0.4**, dcase2);

**data** CXO_results_A2; merge CXO_results1-CXO_results4 CXO_results4_2; by M ;

**data** CXO_results_B2; merge CXO_results5-CXO_results8 CXO_results8_2; by M variable;

**run**;

[SAS codes 4-3b] Analysis when 1 period=3days

*macros used are dcase [SAS codes 4-1c], SCL [4-1d], VF [4-1e], MH [4-1f] GL [4-1g] GL_bs[4-1h] ,dcase2 [4-2a], SCL2 [4-2b], VF2 [4-2c], MH2 [4-2d], GL2 [4-2e], and GL_bs2 [4-2f];

**data** dpattern3; length ID_subgroup $ **1**;

input ID_subgroup c0-c6;

cards;

A 0 0 0 0 0 0 1

B 0 1 1 0 0 0 0

C 0 0 0 1 1 0 0

D 0 0 0 0 0 1 1

E 1 1 0 0 0 0 0

F 0 0 1 1 0 0 0

G 0 0 0 0 1 1 0

H 1 0 0 0 0 0 0

;

%***dcase***(**10000**,**0.001**,**4**, dpattern3);

%***SCL***(**6**, dcase); %***VF***(**6**, dpattern3, dcase); %***MH***(**6**, dcase); %***GL***(**6**, dcase); %***GL_bs***(**6**, dcase);

**data** dpattern20; set dpattern3; z0=**0**;

**data** dpattern21; set dpattern3; z0=**1**;

**data** dpattern2; set dpattern21 dpattern20;

%***dcase2***(**10000**,**0.001**, **4**, **2**, **0.2**, **0.4**);

%***SCL2***(**6**, **0.2**, **0.4**, dcase2); %***VF2***(**6**, **0.2**, **0.4**, dpattern3, dcase2);

%***MH2***(**6**, **0.2**, **0.4**, dcase2); %***GL2***(**6**, **0.2**, **0.4**, dcase2); %***GL_bs2***(**6**, **0.2**, **0.4**, dcase2);

**data** CXO_results_A3; merge CXO_results1-CXO_results4 CXO_results4_2; by M ;

**data** CXO_results_B3; merge CXO_results5-CXO_results8 CXO_results8_2; by M variable;

**run**;

[SAS codes 4-3c] Analysis when 1 period=4days

*macros used are dcase [SAS codes 4-1c], SCL [4-1d], VF [4-1e], MH [4-1f] GL [4-1g] GL_bs[4-1h] ,dcase2 [4-2a], SCL2 [4-2b], VF2 [4-2c], MH2 [4-2d], GL2 [4-2e], and GL_bs2 [4-2f];

**data** dpattern3; length ID_subgroup $ **1**;

input ID_subgroup c0-c4;

cards;

A 0 1 0 0 0

B 0 0 0 0 0

C 0 0 0 1 1

D 0 1 1 0 0

E 1 0 0 0 0

F 0 0 0 0 1

G 0 0 1 1 0

H 1 0 0 0 0

;

%***dcase***(**10000**,**0.001**,**4**, dpattern3);

%***SCL***(**4**, dcase); %***VF***(**4**, dpattern3, dcase); %***MH***(**4**, dcase); %***GL***(**4**, dcase); %***GL_bs***(**4**, dcase);

**data** dpattern20; set dpattern3; z0=**0**;

**data** dpattern21; set dpattern3; z0=**1**;

**data** dpattern2; set dpattern21 dpattern20;

%***dcase2***(**10000**,**0.001**, **4**, **2**, **0.2**, **0.4**);

%***SCL2***(**4**, **0.2**, **0.4**, dcase2); %***VF2***(**4**, **0.2**, **0.4**, dpattern3, dcase2);

%***MH2***(**4**, **0.2**, **0.4**, dcase2); %***GL2***(**4**, **0.2**, **0.4**, dcase2); %***GL_bs2***(**4**, **0.2**, **0.4**, dcase2);

**data** CXO_results_A4; merge CXO_results1-CXO_results4; by M ;

**data** CXO_results_B4; merge CXO_results5-CXO_results8; by M variable;

**run**;

[SAS codes 4-3d] Analysis when 1 period=7days

*macros used are dcase [SAS codes 4-1c], SCL [4-1d], VF [4-1e], MH [4-1f] GL [4-1g] GL_bs[4-1h] ,dcase2 [4-2a], SCL2 [4-2b], VF2 [4-2c], MH2 [4-2d], GL2 [4-2e], and GL_bs2 [4-2f];

**data** dpattern3; length ID_subgroup $ **1**;

input ID_subgroup c0-c2;

cards;

A 0 1 1

B 0 0 0

C 0 0 0

D 0 0 0

E 1 1 1

F 0 0 0

G 0 0 0

H 1 0 0

;

%***dcase***(**10000**,**0.001**,**4**, dpattern3);

%***SCL***(**2**, dcase); %***VF***(**2**, dpattern3, dcase); %***MH***(**2**, dcase); %***GL***(**2**, dcase); %***GL_bs***(**2**, dcase);

**data** dpattern20; set dpattern3; z0=**0**;

**data** dpattern21; set dpattern3; z0=**1**;

**data** dpattern2; set dpattern21 dpattern20;

%***dcase2***(**10000**,**0.001**, **4**, **2**, **0.2**, **0.4**);

%***SCL2***(**2**, **0.2**, **0.4**, dcase2); %***VF2***(**2**, **0.2**, **0.4**, dpattern3, dcase2);

%***MH2***(**2**, **0.2**, **0.4**, dcase2); %***GL2***(**2**, **0.2**, **0.4**, dcase2); %***GL_bs2***(**2**, **0.2**, **0.4**, dcase2);

**data** CXO_results_A7; merge CXO_results1-CXO_results4 CXO_results4_2; by M ;

**data** CXO_results_B7; merge CXO_results5-CXO_results8 CXO_results8; by M variable;

**run**;

[SAS codes 4-3e] Analysis when 1 period=11days

*macros used are dcase [SAS codes 4-1c], SCL [4-1d], VF [4-1e], MH [4-1f] GL [4-1g] GL_bs[4-1h] ,dcase2 [4-2a], SCL2 [4-2b], VF2 [4-2c], MH2 [4-2d], GL2 [4-2e], and GL_bs2 [4-2f];

**data** dpattern3; length ID_subgroup $ **1**;

input ID_subgroup c0-c1;

cards;

A 0 1

B 0 0

C 0 0

D 0 1

E 1 0

F 0 0

G 0 0

H 1 0

;

%***dcase***(**10000**,**0.001**,**4**, dpattern3);

%***SCL***(**1**, dcase); %***VF***(**1**, dpattern3, dcase); %***MH***(**1**, dcase); %***GL***(**1**, dcase); %***GL_bs***(**1**, dcase);

**data** dpattern20; set dpattern3; z0=**0**;

**data** dpattern21; set dpattern3; z0=**1**;

**data** dpattern2; set dpattern21 dpattern20;

%***dcase2***(**10000**,**0.001**, **4**, **2**, **0.2**, **0.4**);

%***SCL2***(**1**, **0.2**, **0.4**, dcase2); %***VF2***(**1**, **0.2**, **0.4**, dpattern3, dcase2);

%***MH2***(**1**, **0.2**, **0.4**, dcase2); %***GL2***(**1**, **0.2**, **0.4**, dcase2); %***GL_bs2***(**1**, **0.2**, **0.4**, dcase2);

**data** CXO_results_A11; merge CXO_results1-CXO_results4 CXO_results4_2; by M ;

**data** CXO_results_B11; merge CXO_results5-CXO_results8 CXO_results8_2; by M variable;

**run**;

**Appendix 4-4 SAS codes for creating and analyzing simulated data in the example in Background section in the text**

[SAS codes 4-4] SAS codes for the example in Background section in the text

In this part, SAS codes for creating and analyzing data of a hypothetical cyclic treatment mentioned in Background section in the text are given. It is assumed that the population consists of 3 Subgroups A, B, and C which have a cyclic treatment pattern with 2 exposed periods followed by 1 unexposed period with no stoppers and no starters.

**data** dpattern;length ID_subgroup $ **1**;

input ID_subgroup c0 c1 c2 c3 c4 c5 c6 c7 c8 c9;

cards;

A 1 1 0 1 1 0 1 1 0 1

B 1 0 1 1 0 1 1 0 1 1

C 0 1 1 0 1 1 0 1 1 0

;

*The codes above are followed by macros dcase [SAS codes 4-1c] and SCL [4-1d];

%***dcase***(**10000**,**0.001**,**4**, dpattern); %***SCL***(**9**, dcase);**run**;

The results given in data set "CXO_results1" created by %SCL are as follows indicating that OR_SCL is underestimated as 3.17 when M=3. It may be also noted that when the number of (1 case period + M control periods) is the integral multiple of 3 (i.e., M=2, 5, 8, ---), OR_SCL is unbiased.

| M | OR_SCL | OR_SCL_L | OR_SCL_U |
| --- | --- | --- | --- |
| 1 | 4.000 | 2.000 | 7.998 |
| 2 | 4.000 | 2.073 | 7.719 |
| 3 | 3.174 | 1.633 | 6.167 |
| 4 | 3.770 | 1.941 | 7.323 |
| 5 | 4.000 | 2.073 | 7.719 |
| 6 | 3.488 | 1.804 | 6.743 |
| 7 | 3.819 | 1.974 | 7.388 |
| 8 | 4.000 | 2.073 | 7.719 |
| 9 | 3.630 | 1.879 | 7.010 |

**Appendix 5**

**Case-crossover study on the association between celecoxib and peripheral edema: a study using Japanese claims database**

The study was approved by the ethics committee of Tokyo University of Science. Japanese data came from 25 corporate-type health insurance plans provided by Cross-Fact database (formerly known as Medi-Scope®) (<https://www.jmiri.jp/medi_scope> Accessed February 20, 2021). Those who were 75 years old or older were not included in the corporate-type health insurance plan and we used claims data for those who were 20 to 74 years old. Data for 60 months between May 2013 to April 2018 were used (males: N=1,163,968; age (SD)=42.5 (13.2), females: N=1,349,901; age (SD)=42.2 (13.1)). As no enrollment data was available, we identified the first and last monthly claims and defined the study period as the period between the first day of the month when the first claim was issued (the first day of the observation) and the last day of the month when the last claim was issued (the last day of the observation) for all individuals. We examined the association between celecoxib and peripheral edema which was one of 44 ‘positive pairs’ in a previous study conducted to assess the validity of sequence symmetry analysis (Wahab IA, Pratt NL, Wiese MD. Pharmacoepideiol Drug Saf 2013; 22:496-502). The processes for patient selection are depicted in Appendix Figures 5a and 5b. We identified 99,395 new users of celecoxib, defined as those with a dispensing of celecoxib after at least 180 days of non-use following the first day of the observation. As in the previous study, new use of furosemide (dispensing of furosemide after at least 180 days of non-use following the first day of the observation) was used to define the occurrence of peripheral edema. The period between the day of dispensing to the last day of supply (the day of dispensing + days supply -1) was defined as exposed. Day X after the last day of supply was defined as X days after the last day of supply. If the next celecoxib script was dispensed up to 7 days after the last day of supply, use was considered continuous (7-day grace period). Otherwise, the exposed period was considered to end on the last day of supply of the last dispensing and the unexposed period started from the day after the last day of exposure until the day before the next dispensing. We found 1,516 possible cases who were new users of celecoxib as well as new users of furosemide. The index date was defined as the day when the outcome occurred. For the case-crossover study, we excluded 1152 concordant cases who were always exposed or always unexposed during study period of 84 days (the index day plus preceding 83 days) and 53 cases who started celecoxib and furosemide on the same day to obtain 311 cases. 53 cases who started celecoxib and furosemide on the same day were excluded because furosemide might be prescribed for prevention rather than treatment of edema.


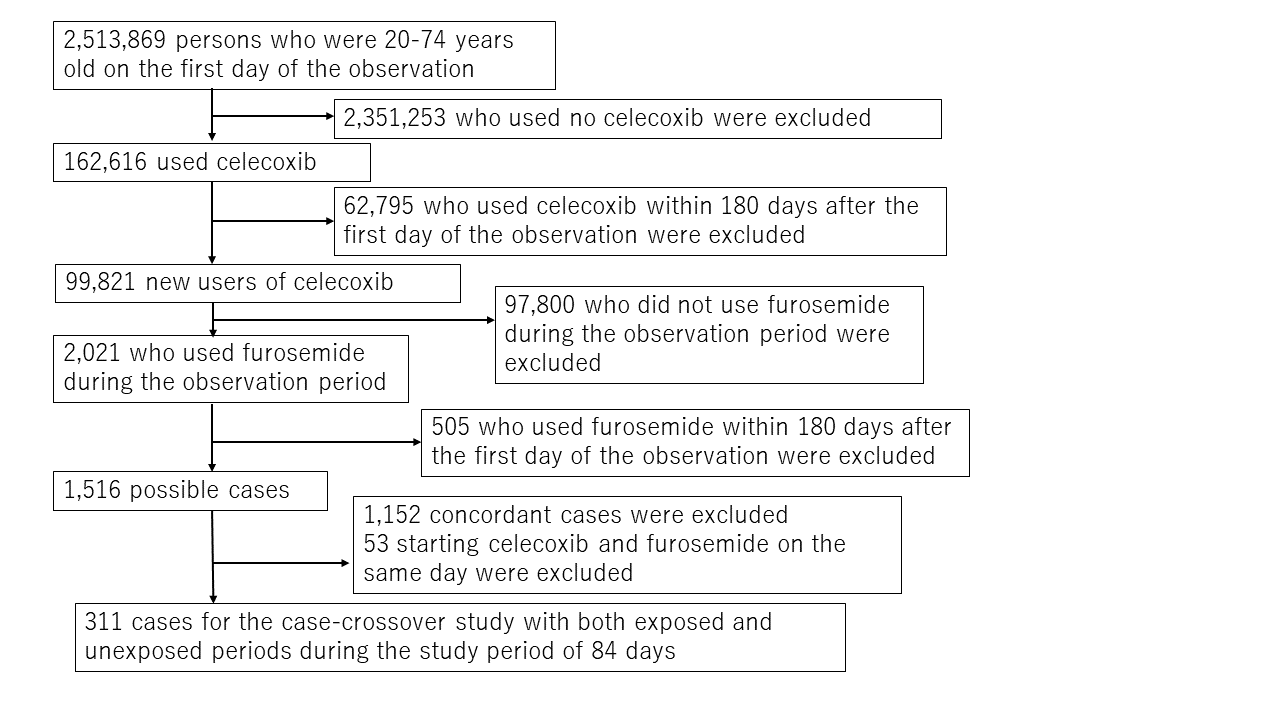


**Appendix Figure 5a** Selection of cases in Japanese study


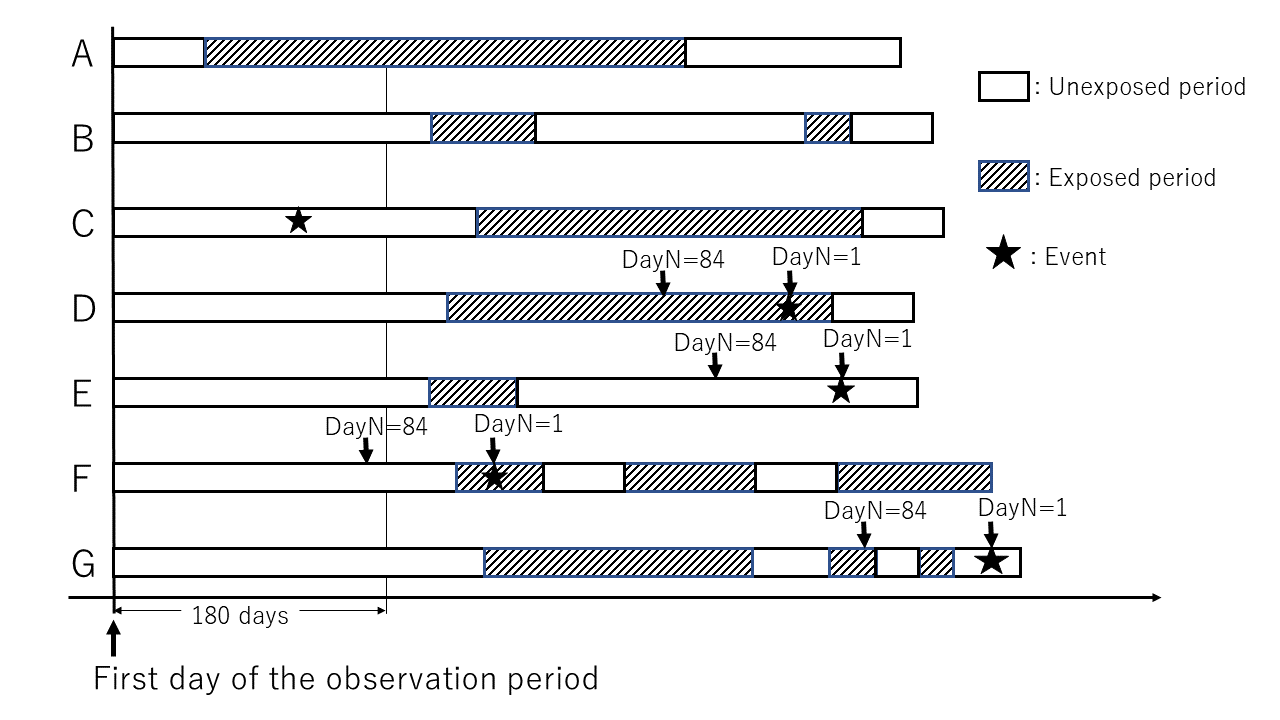


**Appendix Figure 5b** Diagrammatic explanation for potential cases.

**Patient A** was one of 62,795 patients who started celecoxib within 180 days after the first day of the observation period and this patient was excluded; **Patient B** was one of 97,800 patients who first used celecoxib after 180 days of non-use following the first day of the observation period but did not receive furosemide, and was excluded; **Patient C** was one of 505 who were new users of celecoxib but used furosemide within 180 days after the first day of the observation period and this patient was excluded; **Patient D or E** who were one of 1,152 concordant cases were excluded; **Patients F or G** were one of 311 cases in the case-crossover study who had both exposed and unexposed periods during the 84-day study period; **Patient F** was excluded from the additional study where the study period was extended to 336 days while **Patient G** was included in the additional study with a 336-day study periods

For cases, the 84-day study period was divided into (M+1) periods where M=1, 2, 5, 11, 27 and 83 where 1 period included 42, 28, 14, 7, 3 and 1 days, respectively. Four different definitions were used to determine the exposure status in the case period as shown in Table 1 in the text. The case and control periods in a case-crossover study were analyzed by the conditional logistic regression using Equations (1) (2) and (4) as well as by the Mantel-Haenszel method.

All the analyses were performed by SAS 9.4 and data and the SAS codes used to analyze the data will be provided on request.

Results were obtained for the 4 definitions of exposure (Table 1 in the text). Table 6 in the text shows the estimates when study period=84 days and Exposure Definition I is used for M=1, 2, 5, 11, 27 and 83, while those for the other definitions are shown in Appendix Tables 5a below. Table 6 in the text shows the estimates when study period=168 days and 336 days and Exposure Definition I is used, while those for the other definitions are shown in Appendix 5b. In general, ${OR}_{SCL}$ was different from ${OR}_{MH}$ and ${OR}_{G}$ except when M=1, and ${OR}_{SCL}$ estimated from Equation (2) increased with the increase of study period: ${OR}_{SCL}$ was between 1.91 and 2.82 when study period = 84 days (Table 5), between 2.13 and 4.19 when study period = 168 days, and between 3.73 and 7.16 when study period=336 days (Table 6). The point estimate of ${OR}_{G}$ from Equation (4) was always the same as that of ${OR}_{MH}$ as expected. Though ${OR}_{G}$ and ${OR}_{MH}$ were stable as compared with ${OR}_{SCL}$, they tended to increase with study period like ${OR}_{SCL}$: ${OR}_{G}$ and ${OR}_{MH}$ were between 1.91 and 1.99 when study period=84 days (Table 5), between 2.13 and 2.45 when study period =168 days, and between 2.92 and 3.73 when study period=336 days (Table 6).

In general, ${OR}_{SCL}$ varied when M varied as well as when study period varied while ${OR}_{G}$ and ${OR}_{MH}$ were relatively stable. ${OR}_{SCL}$, ${OR}_{MH}$ and ${OR}_{G}$ (particularly ${OR}_{SCL}$) tend to increase when study period is increased from 84 days (Table 5 in the text and Appendix Table 5a) to 336 days (Table 6 in the text and Appendix Table 5b). For Definitions I (Tables 5 and 6) and III (Appendix Tables 5a and 5b), odds ratios tended to increase when M increased while the study period was fixed as 84, 168 or 336 days (except for${OR}_{MH}$ and ${OR}_{G}$ when M=1 and study period=336 days). This increase of odds ratios with the increase of M was observed in the Australian study (Table 7) but was not observed in the simulation study assuming no time trends (Table 4), and may be due to time trends of the exposure. For each different exposure definition (Definition I, II, III and IV), when M=1, ${OR}_{SCL}$, ${OR}_{MH}$ and ${OR}_{G}$ were equal to each other but they differed between definitions, (Definition III (0.86) < Definition I (Tables 5 and 6) (1.51) < Definition II (2.26) < Definition IV (2.84)). When M=1 and the study period=168 days or 336 days, no odds ratios were obtained for Definition IV because all the cases were exposed at the case period and the estimate was infinite (Appendix Table 5b).

**Appendix Table 5a** Estimates of ${OR}_{SCL}$ and ${OR}_{MH}$ (95%CI), and ${OR}_{G}$ (2.5-97.5pct): Japanese data on celecoxib-peripheral edema with study period=84 days and Exposure Definitions II, III, and IV

____________________________________________________________________________________________________________________________

Study period 84 days

____________________________________________________________________________________________________________________________

Days in 1 period 1 day 3 days 7 days 14 days 28 days 42 days

M 83 27 11 5 2 1

___________________________________________________________________________________________________________________________

Definition II

${OR}_{SCL}$ 2.82 (2.14-3.71) 2.83 (2.15-3.73) 2.69 (2.05-3.54) 2.45 (1.87-3.22) 2.34 (1.73-3.16) 2.26 (1.62-3.15)

${OR}_{MH}$ 1.99 (1.58-2.51) 2.01 (1.59-2.54) 2.00 (1.58-2.53) 2.00 (1.57-2.55) 2.07 (1.56-2.74) 2.26 (1.62-3.15)

${OR}_{G}$ 1.99 (1.49-2.65) 2.01 (1.51-2.67) 2.00 (1.49-2.66) 2.00 (1.50-2.66) 2.07 (1.51-2.85) 2.26 (1.64-3.23)

Definition III

${OR}_{SCL}$ 2.82 (2.14-3.71) 2.41 (1.84-3.17) 1.86 (1.42-2.43) 1.35 (1.04-1.76) 0.96 (0.74-1.25) 0.86 (0.66-1.13)

${OR}_{MH}$ 1.99 (1.58-2.51) 1.81 (1.44-2.28) 1.55 (1.23-1.95) 1.25 (0.99-1.57) 0.97 (0.76-1.23) 0.86 (0.66-1.13)

${OR}_{G}$ 1.99 (1.49-2.65) 1.81 (1.37-2.39) 1.55 (1.18-2.03) 1.25 (0.96-1.63) 0.97 (0.74-1.25) 0.86 (0.65-1.13)

Definition IV

${OR}_{SCL}$ 2.82 (2.14-3.71) 3.11 (2.38-4.06) 3.00 (2.32-3.88) 2.78 (2.16-3.58) 2.57 (1.98-3.34) 2.84 (2.11-3.84)

${OR}_{MH}$ 1.99 (1.58-2.51) 2.22 (1.77-2.80) 2.28 (1.81-2.86) 2.27 (1.81-2.86) 2.31 (1.80-2.96) 2.84 (2.11-3.84)

${OR}_{G}$ 1.99 (1.49-2.65) 2.22 (1.70-2.87) 2.28 (1.77-2.97) 2.27 (1.80-2.97) 2.31 (1.76-3.04) 2.84 (2.11-3.84)

M is the number of control periods.

${OR}_{SCL}$: odds ratio by the standard conditional logistic regression; ${OR}_{MH}$: odds ratio by the Mantel-Haenszel method; ${OR}_{G}$: odds ratio by the Greenland's method; 95%CI: 95% confidence interval; 2.5-97.5pct: 2.5 to 97.5 percentiles.

**Appendix Table 5b** Estimates of ${OR}_{SCL}$ and ${OR}_{MH}$ (95%CI), and ${OR}_{G}$ (2.5-97.5pct): Japanese data on celecoxib-peripheral edema with Study period=168 and 336 days and Exposure Definitions II, III, and IV

__________________________________________________________________________________________________________

Study period 168 days 　　　　　　　　336 days

＿＿＿＿＿＿＿＿＿＿＿＿＿＿＿＿＿＿＿＿＿＿＿＿＿ ＿＿＿＿＿＿＿＿＿＿＿＿＿＿＿＿＿＿＿＿＿＿＿＿＿＿＿

Days in 1 period 1 day 14 days 84 days 1 day 　　14 days 168 days

M 167 11 1 335 23 1

__________________________________________________________________________________________________________

Definition II

${OR}_{SCL}$ 4.19 (3.16-5.55) 3.76 (2.85-4.97) 3.11 (2.14-4.53) 7.16 (5.27-9.74) 6.60 (4.87-8.94) 4.04 (2.61-6.26)

${OR}_{MH}$ 2.45 (1.94-3.09) 2.45 (1.93-3.12) 3.11 (2.14-4.53) 2.95 (2.28-3.82) 2.98 (2.29-3.88) 4.04 (2.61-6.26)

${OR}_{G}$ 2.45 (1.87-3.25) 2.45 (1.87-3.28) 3.11 (2.20-4.62) 2.95 (2.14-4.09) 2.98 (2.17-4.13) 4.04 (2.68-6.65)

Definition III

${OR}_{SCL}$ 4.19 (3.16-5.55) 2.33 (1.78-3.05) 1.56 (1.14-2.14) 7.16 (5.27-9.74) 4.18 (3.11-5.61) 2.47 (1.70-3.61)

${OR}_{MH}$ 2.45 (1.94-3.09) 1.77 (1.41-2.22) 1.56 (1.14-2.14) 2.95 (2.28-3.82) 2.33 (1.82-2.99) 2.47 (1.70-3.61)

${OR}_{G}$ 2.45 (1.87-3.25) 1.77 (1.36-2.30) 1.56 (1.15-2.18) 2.95 (2.14-4.09) 2.33 (1.74-3.17) 2.47 (1.72-3.76)

Definition IV

${OR}_{SCL}$ 4.19 (3.16-5.55) 5.13 (3.97-6.63) - 7.16 (5.27-9.74) 9.53 (7.27-12.50) -

${OR}_{MH}$ 2.45 (1.94-3.09) 3.20 (2.55-4.01) - 2.95 (2.28-3.82) 4.22 (3.29-5.42) -

${OR}_{G}$ 2.45 (1.87-3.25) 3.20 (2.50-4.21) - 2.95 (2.14-4.09) 4.22 (3.20-5.85) -

When 1 period is 84 days or more, all cases are exposed at case period when Definition IV is used, and the odds ratios are not estimable.

${OR}_{SCL}$: odds ratio by the standard conditional logistic regression; ${OR}_{MH}$: odds ratio by the Mantel-Haenszel method; ${OR}_{G}$: odds ratio by the Greenland's method. 95%CI: 95% confidence interval; 2.5-97.5pct: 2.5 to 97.5 percentiles.
